# “Times of war and time of uncertain peace”: Narratives of parents of childhood cancer survivors*

**DOI:** 10.1590/1518-8345.7005.4263

**Published:** 2024-08-30

**Authors:** Rhyquelle Rhibna Neris, Ana Carolina Andrade Biaggi Leite, Elizabeth Papathanassoglou, Cristina Garcia-Vivar, Juliana de Souza, Lucila Castanheira Nascimento

**Affiliations:** 1 Universidade de São Paulo, Escola de Enfermagem de Ribeirão Preto, PAHO/WHO Collaborating Centre for Nursing Research Development, Ribeirão Preto, SP, Brazil.; 3 Universidad Pública de Navarra, Facultad de Ciencias de la Salud, Pamplona, Navarra, Spain.; 4 University of Alberta, Faculty of Nursing, Edmonton, Alberta, Canada.; 5 Neurosciences, Rehabilitation & Vision Strategic Clinic Network, Alberta Health Service, Edmonton, Alberta, Canada.

**Keywords:** Survivors, Neoplasms, Child, Adolescent, Parents, Qualitative Research

## Abstract

**Objective:**

to analyze the meaning attributed by parents to the extended and permanent survival of childhood cancer.

**Method:**

qualitative narrative inquiry, developed with parents of adolescents and young adults who survived childhood cancer. Recruitment and data collection involved virtual and in-person approaches. The data were collected through semi-structured interviews. Data were analyzed according to reflective thematic analysis.

**Results:**

a total of ten parents were included in the study. Two thematic narrative syntheses were constructed: “Times of war”; and “Time of uncertain peace”, with their respective sub-themes. The cancer diagnosis marks the beginning of times of war in the parents’ lives. They experience cancer treatment as “highs and lows” with potential threats to their children’s lives. After that, “Time of uncertain peace” are reached, and the balance of the family unit is reestablished. However, the fear of recurrence makes the family peace uncertain, and its maintenance requires constant vigilance and attention to the signs and symptoms of a possible new battle.

**Conclusion:**

the results highlight the experience of being a parent of a childhood cancer survivor and can be applied to develop models of care centered on the survivors’ family.

## Highlights

**(1)** Parents’ experiences in childhood cancer survival are neglected in the literature.

**(2)** The acute treatment for childhood cancer is experienced as a time of war.

**(3)** Long-term survival is experienced as a time of peace by the parents.

**(4)** Fear of recurrence makes parental peace uncertain even in long-term survival.

**(5)** Parents should be included in survivor care.

## Introduction

Childhood cancer is often defined as cancers that occur between birth and 14 years old, but can also include cancers up to age 18 or 19 years old^1^ ^)-(^ ^3^. Treatment options for childhood cancer have improved over recent decades. Despite these advances, it must be considered that the overall survival after five years shows considerable variation by region, starting from 8.1% in East Africa, to 73% in Europe and 60% in South America, and reaching 83.0% in North America^4^. Globally, the estimated survival rate is 37.4% for the same 5 years^4^. This variation highlights health inequalities between different countries^5^. Leukemia survivors are approximately one-third of all cancer survivors under 20 years old^6^.

New definitions of what constitutes a cancer survivor have emerged over the years^7^ ^)-(^ ^8^. Survivors have been variously defined as those first diagnosed with cancer or those living with a cancer diagnosis for 5 years or longer^7^. The first definition of the cancer survivor’s concept was made by Mullan in 1985^9^, a physician and cancer survivor, who presented the three “seasons of survival”^9^, which are: acute survival (period after diagnosis, where the focus is on surviving treatment); extended survival (period after treatment ends, often known as remission, where the focus is on dealing with the short-term treatment consequences); and permanent survival (5 years after the end of the treatment, when the patient is “cured”, but needs to deal with the late effects of cancer and treatments). This is the definition that we adopted in this study^9^.

The family is also considered as a survivor of childhood cancer^7^ ^)-(^ ^8^ ^),(^ ^10^, and is often referred to as the cancer’s “second survivor”. Although parents are not directly biologically affected by cancer, they are certainly impacted functionally, socially, and emotionally by the disease. Parents can experience a series of effects after the treatment of their child’s childhood cancer, psychological suffering and post-traumatic stress disorder, loss of income and financial toxicity due to the high burden of expenses experienced during and even after cancer treatment, fear of cancer recurrence, and the need to deal with the late physical effects that children face after treatment^11^ ^)-(^ ^13^. It is these effects that studies show contribute to a poor quality of life among parents^14^.

However, the parents’ experience is still neglected, and little is known about their experience during the survival of childhood cancer. The literature so far includes mainly studies conducted during active treatment, focusing on experiences during chemotherapy^15^ ^)-(^ ^16^, the end-of-life phase or parental grief^17^ ^)-(^ ^18^ from the unique perspective of the cancer survivor himself/herself^19^ ^)-(^ ^21^, or from the perspective of family caregivers of adult survivors^22^. Therefore, this study aimed to fill this gap of knowledge and highlight the parents’ voices of childhood cancer survivors.

The research question that guided this study was: How do parents of childhood cancer survivors experience the extended and permanent phases of survival? This qualitative study aimed to analyze the meaning attributed by parents to the extended and permanent survival of childhood cancer.

## Methods

### Design and approach

We used a narrative inquiry design, based on Squire’s^23^ methodological approach of finding the narrative centered on experience. Narratives built from the experience-centered model involve movement, succession, progress or sequence, they are generally temporal, and involve the articulation or development of the meaning of the experience lived by the narrator^23^. Therefore, a narrative must contain five essential elements: plot (set of facts); characters (who performs the action); time (duration and time when the story takes place); space (place where the action takes place) and environment (space permeated by socioeconomic, moral and psychological characteristics where the characters live)^24^.

### Setting

A pediatric oncohematology outpatient clinic of a pediatric hospital located in the interior of the state of São Paulo (Brazil). This outpatient clinic has a care service for childhood cancer survivors and patients with benign hematological diseases.

### Participants and eligibility criteria

We employed a purposive sampling to invite parents of adolescent or young adult survivors of childhood cancer. Eligibility criteria included age over 18 years, being a parent of the adolescent or young adult with cancer, and having participated in their care. Adolescents and adults are defined as individuals aged between 15 and 39 years. All surviving children of participants in this study had completed childhood cancer treatment and were in the extended and permanent stages of childhood cancer survival.

Recruitment and data collection for this research were carried out between the months of May 2021 and August 2022. Due to the social restrictions caused by the COVID-19 pandemic, recruitment participants occurred in a hybrid way, through face-to-face and remote recruitment, as detailed below:

- Face-to-face recruitment: this was carried out at the pediatric oncohematology outpatient clinic. The primary investigator identified eligible parents through the list of appointments at the outpatient clinic. Potential participants were contacted on the day of adolescent or young adult survivor’s medical appointment. Parents were invited to participate and were provided with clarifications on the study procedures. Those who agreed to participate signed a written informed consent form. After that, the interview with the participant was scheduled according to the participants’ choice, which could: 1) be in person, at the outpatient clinic, while waiting for medical care; or 2) be online on the online platform of their choice and with which they had greater familiarity (Google Meet, Zoom or WhatsApp video);
- Remote recruitment: done online, through platforms such as Instagram and WhatsApp, and by telephone. When the parent was absent at the time of the face-to-face meeting at the outpatient clinic, the researcher approached the survivor on the day of their medical appointment, before or after their consultation, and explained the objectives and procedures of the research, and when given consent by the survivor, they were asked for the telephone number of their parents. Then, the parent was contacted, and a detailed explanation of the purpose and procedures of the study was provided, as well as how the researcher got access to their telephone contact. Those who indicated interest in participating in the study were provided a link to fill the consent form with Google Forms. Additionally, parents were also invited to participate in the study via Instagram. Participants recruited from Instagram were not receiving follow-up at the pediatric oncohematology outpatient clinic, where the main means of recruitment occurred. Through a social profile that publishes content related to cancer and also reports of cancer survivors public experiences, survivors of childhood cancer were identified. Using Instagram direct messaging, the researcher sent an invitation to potential participants, which contained information on the research objectives and procedures; any questions were also answered at that time. After showing interest in participating in the study, the link to fill in the consent form was sent.

### Data collection

Data collection was carried out by a doctoral student specializing in oncology nursing (first author of the study), with previous experience in collecting qualitative data, and who had no previous relationship with the research participants. Study data were collected through individual online interviews, through video calls via WhatsApp and Google Meet; and face-to-face individual interviews, carried out in a private room in the hospital’s outpatient clinic. A total of eight online interviews and two face-to-face interviews were conducted, one with each participant. In both modalities, semi-structured interviews were carried out, using a pre-established interview script, prepared by the researchers, containing guiding questions, such as: “Can you share the story since the first signs and symptoms of cancer your child experienced?” “Can you share the story in relation to your child’s cancer treatment?” “Have you ever heard of cancer survival?”, “What do you think about this expression?” Also, follow-up questions and prompts were introduced, such as “Could you tell me more about it?” These questions allowed for a story to unfold as the participant chose to tell it. Additionally, the authors used a form to collect data related to characterization of parents and childhood cancer survivors.

Both in person and online interviews were recorded with an external voice recorder, without capturing the participants’ image. The audios of the interviews were transcribed in full to perform data analysis. The transcripts were not returned to the participants. However, the interviews were transcribed by a researcher (P.I.) and fully reviewed by a second researcher for the purposes of verifying accuracy and correcting potential errors. Analysis took place concurrently with data collection. This technique allowed us to end the collection when the data from the interviews began to repeat and no new meaning or code was found, which occurred by interview 10^25^ ^)-(^ ^26^.

### Data treatment and analysis

The narrative method allows the use of different types of data analysis methods, the most common being thematic analysis^27^ ^)-(^ ^28^. Therefore, for this study, the thematic reflective analysis method was used^29^ and the process is described in Figure 1.

**Figure 1:** Data analysis framework. Ribeirão Preto, SP, Brazil, 2022

Stage I	The interviews were transcribed in full. Then, to familiarize themselves with the interviews, two researchers actively read through all the interviews looking for patterns and meanings, and the main ideas for coding were noted.
Stage II	The line-by-line coding of the interviews, using the MAXQDA2020^®(^ ^30^ software, was carried out independently by two authors (first and second authors). In this step, a total of 180 *in vivo* codes were generated. The generated codes identified latent aspects of the data, which identified the characteristics that gave shape and specific meanings to the data. After the initial independent coding, there was discussion among all authors and cross referencing of all codes raised. In this stage, the coding was based on grouping the data collected for subsequent identification of the plot of the narratives.
Stage III	After all the data was already coded, considering the relationship and differences between codes identified in the previous step, two narrative syntheses were constructed inductively. A narrative synthesis has the main characteristic of integrating the experience of all participants, considering their convergences and divergences. Additionally, this maintains the essence of the narrations of the participants to exemplify these experiences. This step was conducted by the first and last author of the study.
Stage IV	In the next phase, based on the narratives, the first and last author inductively identified and named the two interpretive themes.
Stage V	Finally, in the last phase of the reflective thematic analysis, the previously constructed themes were refined, and the themes of the final narratives for the study were defined, as well as the subthemes that structure them. This stage was completed through research team discussion and careful review of the results report. The fifth stage of the analysis ensured the coherence of the data present in the themes.

### Ethical considerations

The study was approved by the ethics committees of the proposing and co-participating institutions, with approval ruling nº 012/2020, of 01/24/2020. Participants received all information related to the research prior to entering the study. Informed consent was obtained from all participants.

### Methodological rigor

The rigor of this study was enhanced through the following considerations^31^ ^)-(^ ^32^: 1) Credibility - data analysis was conducted by a research team, the presentation of the codes, themes and subthemes identified and the presentation of relevant quotes from the narratives. Additionally, excerpts from the narratives are presented to illustrate to the reader that the narratives actually present the meanings that the participants give to their experiences; 2) Transferability - presentation on the method of transparency in the methodological and analytical decision-making processes of the research and description of the sociocultural context of the participants; 3) Reliability - rigorous description of the methodological aspects according to the recommendations of the Consolidated Criteria for Reporting Qualitative Studies - COREQ^33^ and also appropriate time spent engaging with participants; and 4) Confirmability - presentation of the limitations and strengths of the study and the researchers’ reflections during the research process.

## Results

A total of fifteen parents were invited to participate in the study, three of them declined the invitation because they were not available to participate. Two parents accepted the invitation and provided consent when entering the research, but later did not respond to contacts to schedule the online interview. After three unsuccessful attempts to schedule the interview, they were excluded from the study. In the end, ten parents of childhood and adolescent cancer survivors participated in the study, nine were mothers and one was a father, and all attended the process of diagnosis and treatment with the patient. The interviews had a mean duration of 75 minutes, with a minimum time of 31 minutes and a maximum of 136 minutes.

Participants’ age ranged from 37-56 years. Considering that most participants were mothers, through purposive sampling, we sought to also include fathers to learn about the gender perspective and identify possible variations in the survival experience according to gender. Only one father was available to recruit during the recruitment process. The participants’ characteristics is presented in detail in Table 1.

**Table 1:** Characterization of parents and childhood cancer survivors. Ribeirão Preto, SP, Brazil, 2022

Parents’ characteristics	*n*
**Relationship with the adolescent or young adult**	
Mother	9
Father	1
**Age (years)**	
31 - 40	3
41 - 50	6
51 - 60	1
**Gender**	
Female	9
Male	1
**Marital status**	
Single	1
Married	7
Common law	2
**Education**	
Elementary school	4
High school	3
Bachelor’s degree	2
Survivors’ characteristics	*n*
**Age (years)**	
11 - 15	3
16 - 20	5
21 - 25	1
26 - 30	1
**Gender**	
Female	4
Male	6
**Survivors’ diagnosis**	
Leukemias	3
Central Nervous System Tumors	3
Soft tissue sarcoma	1
Osteosarcoma	1
Wilms tumor	1
Lymphoma	1
**Time since the diagnosis**	
2 years	2
3 years	1
4 years	4
5 years or more	3
**Treatments**	
Chemotherapy (Chemo)	4
Surgery	1
Chemo + Radiotherapy	1
Chemo + Surgery	2
Chemo + Radiotherapy + Surgery	1
Radiotherapy + Surgery	1

**Figure 2:**
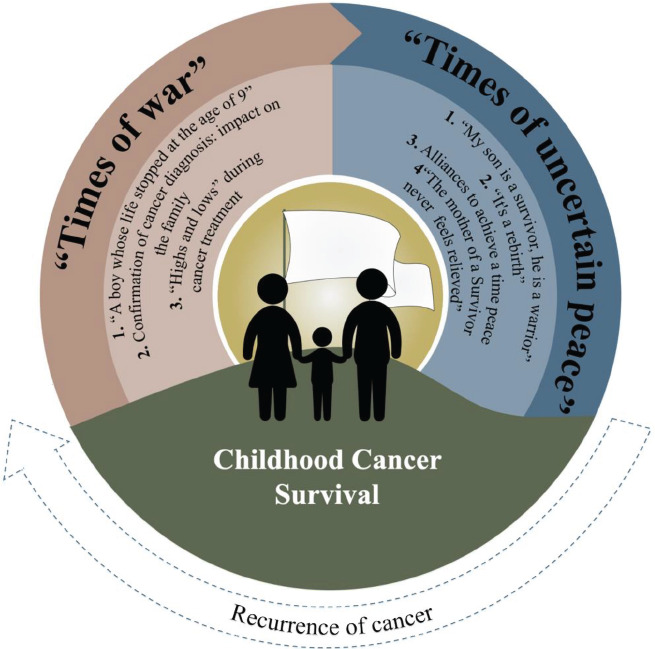
Themes and subthemes synthesis

The results presentation is structured in two main narratives: (1) “Times of war”; and (2) “Times of uncertain peace”, each presenting sub-themes and are illustrated in Figure 2. All of the themes, subthemes and additional participant narratives are listed in Figure 3.

**Figure 3:** Themes, related sub-themes, and selected narratives. Ribeirão Preto, SP, Brazil, 2022

“Times of war”	**Sub-themes**	**Narratives from participants**
	“*A boy whose life stopped at the age of 9*”	*At about 5 years old he started with headaches and vomiting. That went on for several months, he was vomiting and had a headache, I took him to the hospital, they did tests and found nothing. Over time, other symptoms appeared, he found it very difficult to see and speak, his voice was completely choked up, very muffled, sometimes we couldn’t even understand what he was saying* (Mother, p2)*.*
		*On one occasion, he had a very bad seizure, we took him to the hospital, the doctors and health professionals said he was on drugs. I said: No, my son doesn’t use drugs. He is a boy whose life stopped at the age of nine. He doesn’t do drugs. That was the worst day of my life* (Mother, p7).
	Confirmation of cancer diagnosis: impact on the family	*I had no hope for anything else. Honestly, at the time I thought: “I think I’m going to get in my car, go on the road and kill myself with my son”. I think I’m going to kill myself, not just me, but my son. I won’t take it* (Mother, p1).
		*When my daughter was diagnosed, she had severe anemia due to leukemia. She was very much at risk, a 90% chance of death risk, 10% chance of survival* (Father, p4).
	“*Highs and lows*” during cancer treatment	*In order to be able to undergo the treatment, she and I moved to another city and practically lived in the hospital. I didn’t see my daughter, I didn’t see my husband. There was no one who could change place with me to stay with her in the hospital for a while, so I could leave the hospital. There wasn’t anyone. It was a very, very challenging period for me, because I thought I wasn’t going to make it. It wasn’t easy. Not at all. The treatment was very difficult. I thought I was going to go crazy* (Mother, p3).
		*We went through difficult times. She menstruated during treatment and hemorrhaged. Her flow was big, I was desperate. She had a lung water crisis due to the ATRA (trans-retinoic acid) and the saturation dropped to 80%. This time, she was transferred to the ICU (Intensive Care Unit). It was a very difficult time, but she overcame it. There was a time when she caught a cytomegalovirus and was hospitalized for 40 days. There were 25 days without knowing what it was. I thought she was going to die, without finding out, we thought it was the return of the leukemia. There were many highs and lows in the treatment* (Father, p4).
“Time of uncertain peace”	*“My son is a survivor, he is a warrior”*	*There is a time of war and there is a time of peace! Now the time has come for everyone to raise the white flag of peace. Nowadays we just remember what happened and ask God not to have to go through it again, under no circumstances, may God help us so that this does not happen. Now we are a well-structured and balanced family. Today it all has passed. That’s why I say that today is peace time, war time is over. Today, we are winners, for everything that happened; today, we are winners* (Mother, p7).
		*He is a cancer survivor. He survived by not going into a coma, he survived the first surgery, the second surgery, and also the chemo. He survived all of that. He survived three times. He is a survivor, a good survivor!* (Mother, p2)
	*“It’s a rebirth”*	*Today I can see that she is a winner. I say it like this: “you came out of death to be reborn again, it’s a rebirth, a resurgence”. That’s what survival is about, surviving during the critical phase and surviving and being a better person afterwards* (Father, p4).
		*The hospital was an extraordinary thing in my son’s life and brought my son back. He is happy today, he has overcome everything* (Mother, p7).
		*My daughter was born again. God healed her and she didn’t die. She revived again* (Mother, p9)*.*
	Alliances to achieve peacetime	*To be a survivor, you need your family to not give up on treatment. If he hadn’t had the support from me, his father, and other family members and friends, he would have given up* (Mother, p3).
		*My whole family survived cancer. The whole family is a survivor, the family is tied together with the family bonds. When one member goes through something, the family goes through it all together. For all that we’ve been through, surely, we are all cancer survivors* (Mother, p7).
	*“The mother of a survivor never feels relieved”*	*It’s very scary. Very scary. Every time we go for follow-up, we feel that fear. It is terrifying to arrive at the hospital and say something. As much as we have faith in God and we believe it won’t come back, it’s something that haunts me a lot. I don’t know about him, I also rather not put anything in his head so I don’t have to worry about it, with something that is not even happening. Leave it with me, I’m a mother* (Mother, p3).
		*Even though she is fine, there is still that insecurity running around. I still don’t feel safe. This affects my quality of life a little; you stop sleeping and keep thinking. Sometimes I am quiet and thoughtful; suddenly the memories come back and it scares me* (Mother, p5).

The narrative syntheses show that the diagnosis of cancer in children and adolescents marks the beginning of times of war in the parents’ lives, resulting in an imbalance in the family unit. As in every war, the oncological treatment was narrated by the parents as full of “highs and lows”, with several potential threats to the children’s lives. After the “Times of war”, it is a period of “Times of uncertain peace”. During this phase, the balance of the family unit is reestablished and the childhood cancer survival phase begins. Although the war has ended, the parents described that the fear of recurrence is a factor that constantly disturbed the family peace, making it uncertain. Therefore, peace was described as precarious and uncertain, since maintenance of peace requires constant vigilance from parents, who are always attentive to the signs and symptoms of a possible new war.

### 1. Theme: “Times of war”

1. Subtheme - *“A boy whose cancer stopped his life at the age of 9”:* Times of war stopped the lives of its affected. In the same way, parents narrated that their children’s lives were stopped at the pre-cancer diagnosis period. Parents narrate a series of physical symptoms that deviate from everyday normality and alert them to the need to seek answers and treatment for their children. The pre-diagnosis phase is marked by comings and goings in health institutions, which in some cases lasted several months and even years.

One mother described that the symptomatic epilepsy that her son had been presenting, due to the brain tumor, was for a while interpreted as a psychiatric disorder and drug abuse by health professionals. The lived experience of seeking a diagnosis is narrated by the parents through the analogy of “times of war”, due to all the changes in the daily life of the child and the whole family, resulting in an imbalance in the family unit: *It took a while for him to arrive at the diagnosis, he started having these seizures since he was 9 years old. He was 9, 10, 11, 12, 13, 14, 15... he was turning 16. It was many years, many, many years even for the diagnosis. My life stopped from the moment he started the seizures. So, it shook the family, the whole family was unbalanced. Wow, that was wartime!! (Mother, p7)*

Being at war requires stopping life and focusing on one goal: “winning the war”. For this, parents narrated how life had changed since the first signs of cancer.

2. Subtheme - Confirmation of cancer diagnosis: impact on the family*:* After the long journey of reaching a diagnosis, the confirmation of cancer is experienced as a “horrible” moment, of “despair”, a feeling of “the ground disappears” and a “blow”. The stigma of cancer leads parents to think that it is a diagnosis of death. *My son is going to die, is what comes to mind. Today it’s very difficult to see that I survived cancer because it is really aggressive and frightens everyone. I’ve been living one day at a time. It was so awful! I was scared to death of hearing them call my son’s name over the loudspeaker to go into the office. I was really in a desert” (Mother, p1).*

The child’s cancer diagnosis brought on the feeling of “wanting to die” and suicidal ideations, due to the despair of the news and the lack of support felt at the time. The narratives show that the parents enter in a hopelessness cycle that prevents them from believing that their children would respond well to the oncological treatment that was about to begin and would have a good prognosis.

3. Subtheme - *“Highs and lows” during cancer treatment:* The period of treatment was marked by the difficulty in accessing cancer centers to treat the child’s cancer and the geographic separation from the family unit, necessary for the sick child to undergo treatment. For some of the interviewees, mothers assumed the role of caregiver of the sick child and fathers took care of healthy siblings. In addition, the narratives show that many parents had to give up their jobs in order to be able to accompany the child to hospitalizations and meet the treatment demands.

Like any war that is full of battles, the treatment was narrated by parents as permeated by “highs and lows”. In order to “fight cancer”, children and adolescents underwent different types of treatment, such as: chemotherapy, surgery, immunotherapy, and radiotherapy. Parents described several side effects of these treatments that culminated in serious, potentially life-threatening complications, including the need for admission to an intensive care unit. The possibility of being alive at the end of the treatment process and being a survivor was threatened at various times during wartime.

### 2. Theme: “Time of uncertain peace”

1. Subtheme - *“My son is a survivor, he is a warrior”:* After the “times of war”, in which the diagnostic process and the oncological treatment were experienced with highs and lows, and with potential threats to the child’s life, the narratives show that the “times of uncertain peace” arrive, the phase of cancer survival. In times of peace, family balance is restored and the difficult moments experienced during times of war become memories that will forever mark the parents’ lives. Almost unanimously, nine study participants characterize their children as cancer survivors. Only one mother reported not considering her daughter a survivor, since the term referred to something negative and she preferred to use the term “cured” to refer to her daughter. *My son has been through a lot, my son is a survivor! He is a warrior, for all the experience he has gone through and survived. There were many struggles to live through and a lot of grit. When the chemotherapy was finished and he was released to go home, I told the nurses: “My son is a survivor, he is a warrior”. He survived everything! (Mother, p6)*

Participants attributed different terms to characterize their children in addition to the term survivor, these included “winner”, “victorious” and “warrior”. Living after a “diagnosis of death” is an important characteristic in defining survivors from the perspective of parents.

2. Subtheme - *“It’s a rebirth”*: Similar to war, in which survival is uncertain, the treatment of the cancer is like “a near death experience”. Due to the various potential risks to life experienced during “times of war”, survival is experienced as a miracle of life, a rebirth for the survivor. Parents also considered that they had been reborn after the diagnosis and treatment of their children’s cancer. They also reported having new life expectations and priorities after the difficult experience: *It’s a new life. He was born again; I was born again. I had another life expectancy, now it’s another life, totally different (Mother, p8).*

Parents described gratitude for the treatment received at the hospital and, according to them, the hospital brought their child back to life and enabled them to be survivors of childhood cancer.

3. Subtheme - Alliances to achieve peacetime*:* During times of war, it is necessary to form alliances to achieve peace, alliances that help to win each battle necessary to successfully achieve survival. Alliances come from resources such as spirituality, family and friends. In relation to spirituality, faith and hope were used as coping resources, which helped the parents to believe in the reach of times of peace. Another resource mentioned was the family. According to a parent, “to be a survivor, you need your family to not give up”. A mother reported that cancer is a family disease and that it affects the entire unit, therefore, the entire family is survivors of child and adolescent cancer.
4. Subtheme - *“The mother of a survivor never feels relieved”:* As in war, when its end is announced, there is still a doubt about peacetime. Peacekeeping requires constant vigilance and always being aware of the signs and symptoms of a possible new war. Parents described the fear of recurrence as a factor that constantly disturbed the peace: *What still affects me is the fear of recurrence. The mother of a survivor never feels relieved anymore (Mother, p5).*

Thus, they question the times of peace, sometimes in silence and in solitude, the parents did not share their fears. The fear of the return of war is a veiled fear, for the permanence and preservation of family peace, and therefore, it is not shared with other members, including the survivor him/herself. The fear of relapse was described as a factor that affects the parents’ quality of life and that among all parents, the mother is the most affected. Peace in surviving childhood cancer is uncertain, not only because of the potential for new recurrence symptoms, but also because of the traumas that fighting a war leaves behind. *Every time you are afraid and anything scares you. You try to lead a normal life like before, but you can’t. It’s always like: “Oh my God in heaven, is it here again?” Today I’m more attentive to every movement she makes, every little sign is an alert and that’s why today I take more care (Mother, p3).*

## Discussion

The current study provided a deep understanding of the childhood cancer suvivor’s parents’ experiences, relating their experience in times of war and times of uncertain peace. The first theme presented the parents journey to access an adequate diagnosis for the child, sometimes the symptoms presented by the patient were mistaken by health professionals for other disorders, delaying some diagnoses by years. Brazilian health care is notoriously heterogeneous, and many country regions still lack professional training related to pediatric oncology and adequate infrastructure, which make it difficult to detect early childhood cancer. Specialized cancer care services are more concentrated in large cities and in the more developed country regions, which also contributes to inequality in the treatment of childhood cancer^34^. Thus, these regional differences in the provision of these services mean that the survival means in the country are still below those expected for the development and technical knowledge of the current moment. This happens since issues inherent to the organization of the health system and timely access to diagnosis contribute to determining different chances of survival^34^ ^)-(^ ^35^.

The results showed that parents and their children face multifaceted challenges in the diagnosis, treatment and survival of childhood cancer. The lived experience profoundly marks their lives, in a way that they integrate a new identity after cancer, and that it is not possible to be the same as they were before the disease. Nine of the ten participating parents described their children as survivors of childhood cancer, and also attributed to them and families the identity of “survivor”. The National Cancer Institute’s (NCI) definition of a cancer survivor includes family members, friends, and caregivers, as they are also affected by the cancer^10^ ^),(^ ^36^. However, a parent in this study did not identify his son and his family as survivors and similarly, the literature shows that in fact not everyone identifies with the term survivor^37^ ^)-(^ ^38^. Evidence indicates that the identification of not being a cancer survivor is often influenced by the course of treatment (for example, it does not require chemotherapy and radiation), a favorable prognosis, as well as trust in cancer care providers^39^ ^)-(^ ^40^.

Living after cancer is a persistently “disruptive” experience^41^ and even in long-term survival, the levels of parental stress triggered by diagnosis and treatment may not decrease^42^ ^)-(^ ^43^. This study’s results showed that the fear experienced by parents affects their peace in survival and also their quality of life. Evidence shows elevated levels of psychological distress such as post-traumatic stress symptoms, depression, anxiety, sleep disturbances, somatic symptoms, fear of recurrence, extensive worrying, and fatigue among parents of childhood cancer survivors^42^ ^)-(^ ^44^. Uncertainty after treatment and the need to deal with the unknown leads parents to a hypervigilance cycle and, therefore, continuous suffering^45^. In line with this study’s results, the literature shows that mothers are the family members who feel most affected by psychological distress^46^ ^)-(^ ^47^.

Despite this, the narratives describe that the parents believed that after the “rebirth”, they perceived themselves as better people and so did their children, with new expectations of life. Positive outcomes can also occur after a stressful event, such as a child’s cancer diagnosis^48^, a process defined in the literature as post-traumatic growth^48^ ^)-(^ ^49^. Post-traumatic growth involves the processes of making new meanings after a traumatic event, which may include the discovery of benefits and personal development^48^ ^)-(^ ^49^.

This study presents strengths, which include the narrative method, rigorous description of participant recruitment, semi-structured, comprehensive, and dense interviews, rigorous data analysis with two independent coders, and construction of themes and subthemes with the entire research team. Additionally, the results presented in this study shed light on the experience of being a parent of a childhood cancer survivor and draw attention to the need for future studies, and that health professionals and the media need to be sensitive to this experience, which is still poorly understood in the literature. However, the study’s limitations are recognized. Conducting a single interview with parents may have limited the depth of understanding important aspects of the experience described by participants in the first interview. Parents in two different stages of childhood cancer survival, extended and permanent, were included, which may have limited the understanding of the singularities of the experience of being a parent in each of these survival stages. Additionally, although in other studies that include parents the representation of mothers is higher than that of fathers, including only one father in the sample of this study must also be recognized as a limitation.

This study paves the way for future research with parents of childhood cancer survivors. It is important that future studies explore the experience with different family structures and cultural contexts from that of the participants included in this study, in order to further discuss the narratives of the parents described here and add new insights about the experience lived by them during survival. Although the study was developed during the COVID-19 pandemic, it was not our objective to explore the influence of the COVID-19 pandemic on the experience of these parents. Future qualitative studies can explore the influence of the COVID-19 pandemic on the experience of parents of childhood cancer survivors. In addition, future research should develop models of family-centered survival care, with the aim of minimizing the impact of cancer on their lives. The current care model for surviving cancer in children and adolescents does not yet include the family, the focus is on the patient. In the education area, the perspective presented in this study’s results can guide undergraduate and graduate teaching, training health professionals to be more sensitive to the particularities of the childhood cancer survivors’ parents’ experiences. Future research may also look for possible differences in experiences between fathers and mothers of childhood cancer survivors. This is interesting data for nurses to tailor their care, also taking into account the gender issue in nursing care beyond cancer.

Finally, this study’s results have a series of clinical implications for the scope of nursing, regardless of the performance scenario, specialized or basic care. Primarily, it is necessary for health managers to invest in professional qualification to enable the timely recognition of signs and symptoms of childhood cancer and subsequently reduce the diagnosis time. Nurses can act to identify signs and symptoms of cancer in children and adolescents, contributing to faster access to medical care. As discussed earlier, timely diagnosis increases survival rates from childhood cancer. In this study, parents used metaphors to report their experiences in relation to their children’s childhood cancer survival. Metaphors have the potential to contribute to qualified nursing care, centered on the patient and the family, as they help translate the disease experience and make it accessible to the survivor and their parents^50^. The metaphor system can also contribute to creating other meanings and concepts related to cancer, which is still stigmatized and seen as a diagnosis of death. However, it should be used with respect and sensitivity, observing the form of communication chosen by the family and patient. The results also showed that the fear of recurrence has an impact on parent peace, including the quality of life of parents. Health professionals can consider the importance of offering spaces for parents to express their fears and feelings, as well as information related to childhood cancer recurrence, thus mitigating the impact of this fear on their lives. Unfortunately, much child and adolescent survival care focuses on clinical care, without paying attention to the importance of the psycho-emotional approach.

## Conclusion

These studies’ results bring a new perspective that shows that the experience of parents during childhood cancer survival is permeated by times of war and times of uncertain peace. The results of this research have the potential to provide a deeper and more coherent understanding of the childhood cancer survivors’ parents’ experiences in the extended and permanent survival as well as helping to configure the best nursing evidence-based care for them. Moreover, this knowledge can be used by nurses to develop models of family-centered care for survivors and improve in the long-term the quality of life for parents as well as the survivors themselves.
