# Supplementary material for: “Times of war and time of uncertain peace”: Narratives of parents of childhood cancer survivors
Source: Rev Lat Am Enfermagem. 2024 Aug 30;32:e4263. doi: 10.1590/1518-8345.7005.4263 (PMC11368071; doi:10.1590/1518-8345.7005.4263)
Supplement: Translation

# Translation: “Tempos de guerra e tempos de paz incerta”: narrativas de pais de sobreviventes de câncer infantojuvenil*

**DOI:** 10.1590/1518-8345.7005.4264

**Authors:** Rhyquelle Rhibna Neris, Ana Carolina Andrade Biaggi Leite, Elizabeth Papathanassoglou, Cristina Garcia-Vivar, Juliana de Souza, Lucila Castanheira Nascimento

**Affiliations:** 1 Universidade de São Paulo, Escola de Enfermagem de Ribeirão Preto, Centro Colaborador da OPAS/OMS para o Desenvolvimento da Pesquisa em Enfermagem, Ribeirão Preto, SP, Brasil.; 3 Universidad Pública de Navarra, Facultad de Ciencias de la Salud, Pamplona, Navarra, Espanha.; 4 University of Alberta, Faculty of Nursing, Edmonton, Alberta, Canadá.; 5 Neurosciences, Rehabilitation & Vision Strategic Clinic Network, Alberta Health Service, Edmonton, Alberta, Canadá.

**Keywords:** Sobreviventes, Neoplasias, Criança, Adolescente, Pais, Pesquisa Qualitativa

## Abstract

**Objetivo:**

analisar os significados atribuído pelos pais à sobrevivência estendida e permanente ao câncer infantojuvenil.

**Método:**

estudo qualitativo narrativo, desenvolvido com pais de adolescentes e adultos jovens que sobreviveram ao câncer infantojuvenil. O recrutamento e a coleta de dados envolveram abordagens virtuais e presenciais. Os dados foram coletados por meio de entrevistas semiestruturadas e analisados segundo análise temática reflexiva.

**Resultados:**

um total de dez pais foram incluídos no estudo. Foram construídas duas sínteses narrativas temáticas: “Tempos de guerra”; e “Tempo de paz incerta”, com seus respectivos subtemas. O diagnóstico de câncer marca o início de tempos de guerra na vida dos pais. Eles experienciam o tratamento do câncer como “altos e baixos”, com ameaças potenciais à vida dos seus filhos. Depois disso, chega-se ao “Tempo de paz incerta” e o equilíbrio da unidade familiar é restabelecido. Porém, o medo da recidiva torna a paz familiar incerta, e sua manutenção exige vigilância constante e atenção aos sinais e sintomas de uma possível nova batalha.

**Conclusão:**

os resultados destacam a experiência de ser pai de um sobrevivente de câncer infantil e podem ser aplicados para desenvolver modelos de cuidado centrados na família dos sobreviventes.

## Destaques

**(1)** As experiências dos pais na sobrevivência ao câncer infantil ainda são negligenciadas na literatura.

**(2)** O tratamento agudo do câncer infantil é experienciado como um tempo de guerra.

**(3)** A sobrevivência a longo prazo é experienciado como um momento de paz pelos pais.

**(4)** O medo da recidiva torna a paz dos pais incerta, mesmo na sobrevivência a longo prazo.

**(5)** Os pais devem ser incluídos nos cuidados aos sobreviventes.

## Introdução

O câncer infantojuvenil é frequentemente definido como câncer que ocorre entre o nascimento e os 14 anos de idade, mas também pode incluir cânceres até os 18 ou 19 anos^1^ ^)-(^ ^3^. As opções de tratamento para o câncer infantojuvenil melhoraram nas últimas décadas. Apesar desses avanços, deve-se considerar que a sobrevida global após cinco anos apresenta variação considerável por região, começando de 8,1% na África Oriental, a 73% na Europa e 60% na América do Sul, e chegando a 83,0% na América do Norte^4^. Globalmente, a taxa de sobrevivência estimada é de 37,4% para os mesmos 5 anos^4^. Essa variação evidencia desigualdades em saúde entre diferentes países^5^. Os sobreviventes de leucemia representam aproximadamente um terço de todos os sobreviventes de câncer com menos de 20 anos^6^.

Novas definições sobre o que constitui um sobrevivente de câncer surgiram ao longo dos anos^7^ ^)-(^ ^8^. Os sobreviventes têm sido definidos de várias maneiras como aqueles que foram diagnosticados com câncer pela primeira vez ou aqueles que vivem com um diagnóstico de câncer por 5 anos ou mais^7^. A primeira definição do conceito de sobrevivente do câncer foi feita por Mullan em 1985^9^, médico e sobrevivente do câncer, que apresentou as três “estações de sobrevivência”^9^, que são: sobrevida aguda (período após o diagnóstico, onde o foco está no tratamento de sobrevivência); sobrevivência estendida (período após o término do tratamento, muitas vezes conhecido como remissão, onde o foco está em lidar com as consequências do tratamento a curto prazo); e sobrevida permanente (5 anos após o término do tratamento, quando o paciente está “curado”, mas precisa lidar com os efeitos tardios do câncer e dos tratamentos). Esta é a definição que adotamos neste estudo^9^.

A família também é considerada sobrevivente do câncer infantojuvenil^7^ ^)-(^ ^8^ ^),(^ ^10^, sendo muitas vezes referida como a “segunda sobrevivente” do câncer. Embora os pais não sejam biologicamente afetados pelo câncer, são certamente afetados funcionalmente, socialmente e emocionalmente pela doença. Os pais podem vivenciar uma série de efeitos após o tratamento do câncer de seu filho, sofrimento psicológico e transtorno de estresse pós-traumático, perda de renda e toxicidade financeira devido à alta carga de gastos vivenciados durante e mesmo após o tratamento do câncer, medo da recidiva do câncer e a necessidade de lidar com os efeitos físicos tardios que as crianças enfrentam após o tratamento^11^ ^)-(^ ^13^. Os estudos mostram que esses efeitos contribuem para uma má qualidade de vida dos pais^14^.

Contudo, a experiência dos pais ainda é negligenciada e pouco se sabe sobre a experiência deles durante a sobrevivência ao câncer infantojuvenil. A literatura até o momento inclui principalmente estudos realizados durante o tratamento ativo, com foco nas experiências durante a quimioterapia^15^ ^)-(^ ^16^, na fase de fim de vida ou no luto parental^17^ ^)-(^ ^18^ a partir da perspectiva única do próprio sobrevivente do câncer^19^ ^)-(^ ^21^, ou na perspectiva de cuidadores familiares de adultos sobreviventes^22^. Portanto, este estudo teve como objetivo preencher essa lacuna de conhecimento e destacar as vozes dos pais de sobreviventes de câncer infantojuvenil.

A questão de pesquisa que norteou este estudo foi: Como os pais de sobreviventes de câncer infantojuvenil vivenciam as fases estendida e permanente da sobrevivência? Este estudo qualitativo teve como objetivo analisar os significados atribuídos pelos pais à sobrevivência estendida e permanente ao câncer infantojuvenil.

## Método

### Delineamento

Trata-se de uma pesquisa qualitaitva narrativa, baseado na abordagem metodológica de Squire^23^ de narrativa centrada na experiência. As narrativas construídas a partir do modelo centrado na experiência envolvem movimento, sucessão, progressão ou sequência, são geralmente temporais e envolvem a articulação ou desenvolvimento do significado da experiência vivida pelo narrador^23^. Uma narrativa deve conter cinco elementos essenciais: enredo (conjunto de fatos); personagens (quem realiza a ação); tempo (duração e horário em que a história se passa); espaço (local onde acontece a ação) e ambiente (espaço permeado pelas características socioeconômicas, morais e psicológicas onde os personagens vivem)^24^.

### Local do estudo

Ambulatório de oncohematologia pediátrica de um hospital pediátrico localizado no interior do estado de São Paulo (Brasil). Este ambulatório conta com serviço de atendimento a sobreviventes de câncer infantojuvenil e pacientes com doenças hematológicas benignas.

### Participantes e critérios de elegibilidade

Empregamos uma amostragem intencional para convidar pais de adolescentes ou adultos jovens sobreviventes de câncer infantojuvenil. Os critérios de elegibilidade incluíram: idade superior a 18 anos; ser pai do adolescente ou jovem adulto com câncer e ter participado do seu cuidado. Adolescentes e adultos são definidos como qualquer indivíduo com idade entre 15 e 39 anos. Todos os filhos sobreviventes dos participantes deste estudo completaram o tratamento do câncer infantil e estavam nos estágios de sobrevivência estendidas e permanente ao câncer infantil.

O recrutamento e a coleta de dados para esta pesquisa foram realizados entre os meses de maio de 2021 e agosto de 2022. Devido às restrições sociais causadas pela pandemia de COVID-19, o recrutamento dos participantes ocorreu de forma híbrida, por meio de recrutamento presencial e remoto, conforme detalhado abaixo:

- Recrutamento presencial: realizado no ambulatório de oncohematologia pediátrica. A investigadora principal identificou os pais elegíveis através da lista de consultas no ambulatório. Os potenciais participantes foram contatados no dia da consulta médica do adolescente ou jovem adulto sobrevivente. Os pais foram convidados a participar e receberam esclarecimentos sobre os procedimentos do estudo. Aqueles que concordaram em participar assinaram um termo de consentimento livre e esclarecido por escrito. Após isso, a entrevista com o participante foi agendada conforme escolha dos participantes, que poderia ser: 1) presencial, no ambulatório, enquanto aguardava atendimento médico; ou 2) *online* na plataforma de sua preferência e com a qual tivesse maior familiaridade (*Google Meet*, *Zoom* ou *WhatsApp* vídeo);
- Recrutamento remoto: feito on-line, através de plataformas como *Instagram* e *WhatsApp*, e por telefone. Quando o responsável estava ausente no momento do encontro presencial no ambulatório, o pesquisador abordava o sobrevivente no dia da consulta médica, antes ou depois da consulta, e explicava os objetivos e procedimentos da pesquisa, e quando dado o consentimento do sobrevivente, foi solicitado o número de telefone dos pais. Em seguida, os pais foram contatados e uma explicação detalhada do objetivo e dos procedimentos do estudo era fornecida, bem como a forma como a pesquisadora obteve acesso ao seu contato telefônico. Aqueles que manifestaram interesse em participar do estudo receberam um link para preenchimento do termo de consentimento no Formulários Google. Além disso, os pais também foram convidados a participar do estudo via Instagram. Os participantes recrutados no Instagram não estavam em acompanhamento no ambulatório de oncohematologia pediátrica, onde ocorreu o principal meio de recrutamento. Por meio de um perfil social que publica conteúdos relacionados ao câncer e também relatos de experiências públicas de sobreviventes, foram identificados sobreviventes de câncer infantojuvenil. Por meio de mensagens diretas do Instagram, a pesquisadora enviou um convite aos potenciais participantes, que continha informações sobre os objetivos e procedimentos da pesquisa; quaisquer dúvidas também foram respondidas naquele momento. Após manifestação de interesse em participar do estudo, foi enviado o link para preenchimento do Termo de Consentimento Livre e Esclarecido.

### Coleta de dados

A coleta de dados foi realizada por uma doutoranda com especialização em enfermagem oncológica (primeira autora do estudo), com experiência prévia em coleta de dados qualitativos, e que não possuía vínculo prévio com os participantes da pesquisa. Os dados do estudo foram coletados por meio de entrevistas individuais on-line, através de videochamadas via *WhatsApp* e *Google Meet*; e entrevistas individuais presenciais, realizadas em sala privativa do ambulatório do hospital. Foram realizadas oito entrevistas on-line e duas entrevistas presenciais, uma com cada participante. Em ambas as modalidades foram realizadas entrevistas semiestruturadas, utilizando um roteiro de entrevista pré-estabelecido, elaborado pelas pesquisadoras, contendo questões norteadoras, como: “Você pode contar a história desde os primeiros sinais e sintomas de câncer do seu filho? ”, “Você pode contar a história em relação ao tratamento do câncer do seu filho?”, “Você já ouviu falar em sobrevivência ao câncer?”, “O que você acha dessa expressão?”. Além disso, foram introduzidas perguntas de acompanhamento, como: “Você poderia me contar mais sobre isso?”. Essas perguntas permitiram que uma história se desenrolasse conforme o participante escolhesse contá-la. Além disso, os autores utilizaram um formulário para coletar dados relacionados à caracterização de pais e sobreviventes de câncer infantil.

Tanto as entrevistas presenciais quanto as on-line foram gravadas com gravador de voz externo, sem captação da imagem dos participantes. Os áudios das entrevistas foram transcritos na íntegra para realização da análise dos dados. As transcrições não foram devolvidas aos participantes. Contudo, as entrevistas foram transcritas por um pesquisador (P.I.) e revisadas na íntegra por um segundo pesquisador para fins de verificação de veracidade e correção de possíveis erros. A análise ocorreu concomitantemente à coleta de dados. Essa técnica permitiu encerrar a coleta quando os dados das entrevistas começaram a se repetir e nenhum novo significado ou código foi encontrado, o que ocorreu na entrevista 10^25^ ^)-(^ ^26^.

### Tratamento e análise de dados

O método narrativo permite a utilização de diferentes tipos de métodos de análise de dados, sendo o mais comum a análise temática^27^ ^)-(^ ^28^. Portanto, para este estudo foi utilizado o método de análise reflexiva temática^29^ e o processo está descrito na Figura 1.

**Figura 1:** Estrutura de análise de dados. Ribeirão Preto, SP, Brasil, 2022

Estágio I	As entrevistas foram transcritas na íntegra. Em seguida, para se familiarizarem com as entrevistas, dois pesquisadores leram ativamente todas as entrevistas em busca de padrões e significados, e as principais ideias para codificação foram anotadas.
Estágio II	A codificação linha por linha das entrevistas foi realizada, de forma independente, por dois autores (primeira e penúltima autoras), por meio do *software* MAXQDA2020^®(^ ^30^. Nesta etapa foram gerados um total de 180 códigos *in vivo*. Os códigos gerados identificavam aspectos latentes dos dado e características que davam forma e significados específicos aos dados. Após a codificação inicial independente, houve discussão entre todos os autores e cruzamento de todos os códigos levantados. Nesta etapa, a codificação baseou-se no agrupamento dos dados coletados para posterior identificação do enredo das narrativas.
Estágio III	Após todos os dados já terem sido codificados, considerando as relações e diferenças entre os códigos identificados na etapa anterior, foram construídas indutivamente duas sínteses narrativas. Uma síntese narrativa tem como principal característica integrar a experiência de todos os participantes, considerando suas convergências e divergências. Além disso, mantém a essência das narrativas dos participantes para exemplificar essas experiências. Esta etapa foi conduzida pelo primeiro e último autor do estudo.
Estágio IV	Na fase seguinte, com base nas narrativas, o primeiro e o último autor identificaram e nomearam indutivamente os dois temas interpretativos.
Estágio V	Por fim, na última fase da análise temática reflexiva, foram refinados os temas previamente construídos, e os temas das narrativas finais do estudo foram definidos, bem como os subtemas que os estruturam. Esta etapa foi concluída através da discussão da equipe de pesquisa e da revisão cuidadosa do relatório de resultados. A quinta etapa da análise também garantiu a coerência dos dados presentes nos temas.

### Considerações éticas

O estudo foi aprovado pelos comitês de ética da instituição proponente e coparticipante, com parecer nº 012/2020, de 24/01/2020. Os participantes receberam todas as informações relacionadas à pesquisa antes de entrar no estudo. O consentimento informado foi obtido de todos os participantes.

### Rigor metodológico

O rigor deste estudo foi potencializado pelas seguintes considerações^31^ ^)-(^ ^32^: 1) Credibilidade - a análise dos dados foi realizada por uma equipe de pesquisa, a apresentação dos códigos, temas e subtemas identificados e a apresentação de citações relevantes das narrativas. Adicionalmente, são apresentados trechos das narrativas para ilustrar ao leitor que as narrativas realmente apresentam os significados que os participantes dão às suas experiências; 2) Transferibilidade - apresentação no método, de forma transparente, dos processos de tomada de decisão metodológicos e analíticos da pesquisa e descrição do contexto sociocultural dos participantes; 3) Confiabilidade - descrição rigorosa dos aspectos metodológicos de acordo com as recomendações do *Consolidated Criteria for Reporting Qualitative Studies* - COREQ^33^ e tempo adequado gasto no envolvimento com os participantes; e 4) Confirmabilidade - apresentação das limitações e dos pontos fortes do estudo e das reflexões dos pesquisadores durante o processo de pesquisa.

## Resultados

Foram convidados a participar do estudo quinze pais, sendo que três deles recusaram o convite por não terem disponibilidade para participar. Dois pais aceitaram o convite e deram consentimento ao ingressar na pesquisa, mas posteriormente não responderam aos contatos para agendamento da entrevista on-line. Após três tentativas de agendamento da entrevista sem sucesso, foram excluídos do estudo. Ao final, participaram do estudo dez pais de crianças e adolescentes sobreviventes de câncer, nove eram mães e um era pai, e todos acompanharam o processo de diagnóstico e tratamento com o paciente. As entrevistas tiveram duração média de 75 minutos, com tempo mínimo de 31 minutos e máximo de 136 minutos.

A idade dos participantes variou de 37 a 56 anos. Considerando que os participantes recrutados e incluídos no estudo eram, predominatemente, mães, por meio da amostra de conveniência, buscou-se incluir um pai para conhecer a perspectiva de gênero e identificar possíveis variações na experiência de sobrevivência de acordo com o gênero. Apenas um pai estava disponível durante o processo de recrutamento. A caracterização dos participantes está apresentada em detalhes na Tabela 1.

**Tabela 1:** Caracterização dos pais e sobreviventes de câncer infantojuvenil. Ribeirão Preto, SP, Brasil, 2022

Características dos participantes	*n*
**Relação com o adolescente ou adulto jovem**	
Mãe	9
Pai	1
**Idade (anos)**	
31 - 40	3
41 - 50	6
51 - 60	1
**Gênero**	
Feminino	9
Masculino	1
**Estado civil**	
Solteiro	1
Casado	7
União estável	2
**Educação**	
Ensino fundamental	4
Ensino médio	3
Ensino superior	2
Caracteristicas dos sobreviventes	*n*
**Idade (anos)**	
11 - 15	3
16 - 20	5
21 - 25	1
26 - 30	1
**Gênero**	
Feminino	4
Masculino	6
**Diagnóstico do sobrevivente**	
Leucemia	3
Tumor de Sistema Nervoso Central	3
Sarcoma de Partes Moles	1
Osteosarcoma	1
Tumor de Wilms	1
Linfomas	1
**Tempo desde o diagnóstico**	
2 anos	2
3 anos	1
4 anos	4
5 anos ou mais	3
**Tratamentos**	
Quimioterapia (QT)	4
Cirurgia	1
Quimioterapia + Radioterapia	1
Quimioterapia + Cirurgia	2
Quimioterapia + Radioterapia + Cirurgia	1
Radioterapia + Cirurgia	1

A apresentação dos resultados está estruturada em duas narrativas principais: (1) “Tempos de guerra”; e (2) “Tempos de paz incerta”, cada um apresentando subtemas e ilustrados na Figura 2. Todos os temas, subtemas e narrativas adicionais dos participantes estão listados na Figura 3.

**Figura 2:** Síntese dos temas e dos subtemas

**Figura 3:** Temas, subtemas e narrativas selecionadas. Ribeirão Preto, SP, Brasil, 2022

“Tempos de guerra”	**Subtemas**	**Narrativas dos participantes**
	“*Um menino que parou a vida com 9 anos*”	*Com uns 5 anos ele começou com uma dor de cabeça e vômito. Isso foram vários e vários meses, ele tendo os vômitos e dor de cabeça, eu levava ele no hospital, eles faziam exames e não encontravam nada. Com o tempo outros sintomas foram aparecendo, ele ficou com muita dificuldade para enxergar e para falar, a voz estava muito embargada, muito embolada, tinha vez que a gente nem entendia o que ele falava* (Mãe, p2).
		*Em uma ocasião, ele teve uma convulsão muito forte, levamos ele para o hospital, os médicos e profissionais de saúde falaram que ele estava drogado. Eu falei: “Não, meu filho não usa droga. Ele é um menino que parou a vida com nove anos. Ele não usa drogas”. Esse foi o pior dia da minha vida* (Mãe, p7).
	Confirmação do diagnóstico de câncer: impacto na família	*Eu não tinha esperança de mais nada. Sinceramente, na hora pensei: “acho que vou entrar no carro, pegar a estrada e me matar com meu filho”. Acho que vou me matar, não só eu, mas meu filho. Eu não vou aguentar isso* (Mãe, p1).
		*Quando minha filha foi diagnosticada, ela tinha anemia grave devido à leucemia. Ela corria muito risco, 90% de chance de risco de morte, 10% de chance de sobreviver* (Pai, p4).
	“*Altos e baixos*” durante o tratamento	*Para conseguir fazer o tratamento, eu e ela mudamos de cidade e passamos a praticamente morar no hospital. Eu não via meu filho, não via meu marido. Não tinha com quem eu trocar para ficar um pouquinho com ela no hospital, para eu sair de dentro do hospital. Não tinha ninguém. Foi um período muito, muito desafiador para mim, porque eu achei que não ia dar conta. Não foi fácil. Não mesmo. Foi muito difícil o tratamento. Eu achei que eu iria ficar louca* (Pai, p3).
		*Passamos por momentos difíceis. Ela menstruou durante o tratamento e teve hemorragia. O fluxo era grande, eu ficava desesperado. Ela teve uma crise de água no pulmão devido ao ATRA (ácido trans-retinóico) e a saturação caiu para 80%. Nessa vez, ela foi transferida para a UTI (Unidade de Terapia Intensiva). Foram momentos muito difíceis, mas que ela se superou. Teve uma vez que ela pegou um citomegalovírus e ficou 40 dias internada. Ficou 25 dias sem saber o que que era. Achei que ela iria morrer, que não descobriria, achamos que era a volta da leucemia. Tiveram muitos altos e baixos do tratamento* (Pai, p4).
“Tempos de paz incerta”	*“Meu filho é um sobrevivente, ele é um guerreiro”*	*Há o tempo de guerra e há o tempo de paz! Agora chegou o momento que todos levantaram a bandeira branca, da paz. Hoje em dia, a gente só recorda o que passou e pede a Deus para que não venha passar por isso de novo, em hipótese alguma, que Deus nos ajude para que isso não aconteça. Agora somos uma família bem estruturada e equilibrada. Hoje tudo passou. Por isso eu falo que hoje é tempo de paz, tempo de guerra acabou. Hoje, nós somos vencedores, por tudo o que aconteceu; hoje, nós somos vencedores* (Mãe, p7).
		*Ele é um sobrevivente do câncer. Ele sobreviveu ao não entrar em coma, sobreviveu à primeira cirurgia, à segunda cirurgia e também à quimio. Ele sobreviveu a tudo isso. Ele sobreviveu umas três vezes. Ele é um sobrevivente bem sobrevivente!* (Mãe, p2).
	*“É um renascimento”*	*Hoje eu consigo ver que ela é uma vitoriosa. Eu falo assim: “você saiu da morte pra renascer de novo, é um renascimento, um ressurgimento”. A sobrevivência é isso, é você sobreviver na fase crítica e você sobreviver e ser uma pessoa melhor depois* (Pai, p4).
		*O hospital foi extraordinário na vida do meu filho e trouxe meu filho de volta. Ele está feliz hoje, ele superou tudo* (Mãe, p7).
		*Minha filha nasceu de novo. Deus a curou e ela não morreu. Ela reviveu novamente* (Mãe, p9).
	Alianças para alcançar os tempos de paz	*Para ser sobrevivente, precisa da família para não desistir do tratamento. Se ele não tivesse o meu apoio, do pai dele e das outras pessoas da família e amigos, ele teria desistido* (Mãe, p3).
		*Minha família toda sobreviveu ao câncer. A família toda é sobrevivente, a família está tudo junto e família são laços. Quando um membro passa por algo, a família passa tudo junto. Por tudo que nós passamos, com toda certeza todos nós somos sobreviventes do câncer (Mãe, p7).*
	*“A mãe de um sobrevivente nunca se sente aliviada”*	*Dá muito medo. Muito medo. Cada vez que vai fazer um exame, a gente fica naquele medo. Dá um apavoramento de chegar no hospital e falar alguma coisa. Por mais que a gente tenha fé em Deus e a gente acredita que não vai voltar, mas é uma coisa que me assombra muito. Da parte dele eu não sei, eu prefiro também nem colocar nada na cabeça dele para não ter que se preocupar com isso, com uma coisa que nem está acontecendo. Deixa para mim, que sou mãe* (Mãe, p3).
		*Mesmo ela estando bem, ainda fica, assim, aquela insegurança rodando. Ainda não senti uma segurança. Isso afeta um pouco a minha qualidade de vida; você deixa de dormir e fica pensando. Às vezes, estou quieta e pensativa; de repente vem as lembranças e isso me assusta* (Mãe, p5).

As sínteses narrativas mostram que o diagnóstico de câncer em crianças e adolescentes marca o início de tempos de guerra na vida dos pais, resultando em desequilíbrio no núcleo familiar. Como em toda guerra, o tratamento oncológico foi narrado pelos pais como cheio de “altos e baixos”, com diversas ameaças potenciais à vida dos filhos. Depois dos “Tempos de guerra”, vem um período de “Tempos de paz incerta”. Nessa fase, o equilíbrio da unidade familiar é restabelecido e inicia-se a fase de sobrevivência ao câncer infantil. Embora a guerra tenha terminado, os pais descreveram que o medo da recidiva é um fator que perturba constantemente a paz familiar, tornando-a incerta. Portanto, a paz foi descrita como precária e incerta, pois a manutenção da paz exige vigilância constante dos pais, que estão sempre atentos aos sinais e sintomas de uma possível nova guerra.

### 1. Tema: “Tempos de Guerra”

1. Subtema - *“Um menino que parou a vida com 9 anos”:* Tempos de guerra interrompem a vida dos afetados por ela. Da mesma forma, os pais narraram que a vida dos filhos foi interrompida no período do diagnóstico pré-câncer. Os pais narram uma série de sintomas físicos que fogem da normalidade cotidiana e os alertam para a necessidade de buscar respostas e tratamento para seus filhos. A fase de pré-diagnóstico é marcada por idas e vindas nas instituições de saúde, que em alguns casos duram vários meses e até anos.

Uma mãe descreveu que a epilepsia sintomática que seu filho vinha apresentando, devido ao tumor cerebral, foi durante algum tempo interpretada como transtorno psiquiátrico e abuso de drogas pelos profissionais de saúde. A experiência vivida na busca pelo diagnóstico é narrada pelos pais através da analogia dos “tempos de guerra”, devido a todas as mudanças no cotidiano da criança e de toda a família, resultando em um desequilíbrio no núcleo familiar: *Demorou para ele chegar no diagnóstico, ele começou a ter essas convulsões desde aos 9 anos. Ele tinha 9, 10, 11, 12, 13, 14, 15... ele estava completando 16 anos já. Foram muitos anos, muitos, muitos anos até o diagnóstico. A minha vida parou a partir do momento que ele começou as convulsões. Então, deu uma balançada na família, um desequilíbrio na família toda. Nossa, foi, assim, tempos de guerra!! (Mãe, p7)*

Estar em guerra exige parar a vida e concentrar-se em um objetivo: “vencer a guerra”. Para isso, os pais narraram como a vida mudou desde os primeiros sinais do câncer.

2. Subtema - Confirmação do diagnóstico de câncer: impacto na família: Após a longa jornada para chegar ao diagnóstico, a confirmação do câncer é vivenciada como um momento “horrível”, de “desespero”, sensação de “falta de chão” e um “baque” . O estigma do câncer leva os pais a pensarem que se trata de um diagnóstico de morte. *Meu filho vai morrer, é o que vem na sua cabeça.. Hoje é muito difícil ver que sobrevivi ao câncer porque é muito agressivo e amedronta todo mundo. Eu fui vivendo um dia de cada vez. Foi horrível! ! Eu morria de medo de ouvir eles chamarem o nome do meu filho no autofalante para entrar no consultório. Eu estava em um deserto mesmo” (Mãe, p1).*

O diagnóstico de câncer do filho despertou o sentimento de “vontade de morrer” e ideações suicidas, devido ao desespero da notícia e falta de apoio sentida na ocasião. As narrativas mostram que os pais entram em um ciclo de desesperança que os impede de acreditar que seus filhos responderiam bem ao tratamento oncológico que estava prestes a ser iniciado e que teriam um bom prognóstico.

3. Subtema - *“Alto e baixos”* durante o tratamento do câncer*:* O período de tratamento foi marcado pela dificuldade de acesso aos centros oncológicos para tratamento do câncer infantojuvenil e pela separação geográfica da unidade familiar, necessária para que a criança doente pudesse realizar o tratamento. Para alguns dos entrevistados, as mães assumiam o papel de cuidadoras do filho doente e os pais cuidavam dos irmãos saudáveis. Além disso, as narrativas mostram que muitos pais tiveram que abrir mão do emprego para conseguir acompanhar o filho às internações e atender às demandas do tratamento.

Como toda guerra repleta de batalhas, o tratamento foi narrado pelos pais como permeado de “altos e baixos”. Para “combater o câncer”, crianças e adolescentes passaram por diversos tipos de tratamento, como: quimioterapia, cirurgia, imunoterapia e radioterapia. Os pais descreveram vários efeitos adversos destes tratamentos que culminaram em complicações graves e potencialmente fatais, incluindo a necessidade de internação na unidade de cuidados intensivos. A possibilidade de estar vivo ao final do processo de tratamento e ser um sobrevivente foi ameaçada em diversos momentos os tempos de guerra.

### 2. Tema: “Tempos de paz incerta”

1. Subtema - *“Meu filho é um sobrevivente, ele é um guerreiro”:* Passados os “tempos de guerra”, em que o processo diagnóstico e o tratamento oncológico foram vivenciados com altos e baixos, e com potenciais ameaças à vida do filho, as narrativas mostram que é chegado os “tempos de paz incerta”, a fase da sobrevivência ao câncer. Em tempos de paz, o equilíbrio familiar é restaurado e os momentos difíceis vividos nos tempos de guerra tornam-se lembranças que marcarão para sempre a vida dos pais. Quase por unanimidade, nove participantes do estudo caracterizam os seus filhos como sobreviventes do câncer. Apenas uma mãe relatou não considerar a filha sobrevivente, pois o termo a referenciava a algo negativo e ela preferiu utilizar o termo “curada” para se referir à filha. *Meu filho já passou por muita coisa, meu filho é um sobrevivente! Ele é um guerreiro, por toda experiência que passou e sobreviveu. Foram muitas lutas para ele conseguir sobreviver e muita garra. Quando terminou a quimioterapia e ele foi liberado para ir para casa, falei para as enfermeiras: “Meu filho é um sobrevivente, é um guerreiro”. Ele sobreviveu a tudo! (Mãe, p6)*

Os participantes atribuíram diferentes termos para caracterizar seus filhos além do termo sobrevivente, estes incluíam “vencedor”, “vitorioso” e “guerreiro”. Viver após um “diagnóstico de morte” é uma característica importante na definição dos sobreviventes na perspectiva dos pais.

2. Subtema - *“*É um renascimento*”*: Semelhante à guerra, em que a sobrevivência é incerta, o tratamento do câncer é como “uma experiência de quase morte”. Devido aos vários riscos potenciais para a vida vivenciados durante “tempos de guerra”, a sobrevivência é vivida como um milagre da vida, um renascimento para o sobrevivente. Os pais também consideraram que haviam renascido após o diagnóstico e tratamento do câncer dos filhos. Também relataram ter novas expectativas e prioridades de vida após a experiência difícil: *É uma vida nova. Ele nasceu de novo; Eu nasci de novo. Eu tinha outra expectativa de vida, agora é outra vida, totalmente diferente (Mãe, p8).*

Os pais expressaram gratidão pelo tratamento recebido no hospital e, segundo eles, o hospital trouxe seu filho de volta à vida e possibilitou que eles fossem sobreviventes do câncer infantil.

3. Subtema - Alianças para alcançar os tempos de paz*:* Em tempos de guerra, é necessário formar alianças para alcançar a paz, alianças que ajudem a vencer cada batalha necessária para alcançar a sobrevivência com sucesso. As alianças vêm de recursos como espiritualidade, família e amigos. Em relação à espiritualidade, a fé e a esperança foram utilizadas como recursos de enfrentamento, o que ajudou os pais a acreditarem no alcance de tempos de paz. Outro recurso mencionado foi a família. Segundo um dos pais, “para ser um sobrevivente, é preciso que a sua família não desista”. Uma mãe relatou que o câncer é uma doença familiar e que atinge toda a unidade, portanto, toda a família é sobrevivente do câncer infantojuvenil.
4. Subtema - *“A mãe de um sobrevivente nunca se sente aliviada”:* Tal como na guerra, quando o seu fim é anunciado, ainda existe dúvidas sobre os tempos de paz. A manutenção da paz exige vigilância constante e estar sempre atento aos sinais e sintomas de uma possível nova guerra. Os pais descreveram o medo da recidiva como um fator que perturbava constantemente a paz: *O que ainda me afeta é o medo da recidiva. A mãe do sobrevivente nunca mais se sente aliviada (Mãe, p5).*

Assim, questionam os tempos de paz, às vezes no silêncio e na solidão, os pais não partilhavam dos seus medos. O medo do retorno da guerra é um medo velado, pela permanência e preservação da paz familiar e, portanto, não é compartilhado com os demais membros, inclusive com o próprio sobrevivente. O medo da recidiva foi descrito como um fator que afeta a qualidade de vida dos pais e que entre todos os pais a mãe é a mais afetada. A paz na sobrevivência ao câncer infantil é incerta, não só devido à possibilidade de novos sintomas de recidiva, mas também devido aos traumas que a luta numa guerra deixa para trás. *Toda vez que você está com medo e alguma coisa te assusta. Você tenta levar uma vida normal como antes, mas não consegue. É sempre tipo: “Meu Deus do céu, está aqui de novo?” Hoje estou mais atenta a cada movimento que ela faz, cada sinalzinho é um alerta e por isso hoje tomo mais cuidado (Mãe, p3).*

## Discussão

O presente estudo proporcionou uma compreensão profunda das experiências dos pais dos sobreviventes do câncer infantil, relacionando a experiência deles em tempos de guerra e em tempos de paz incerta. O primeiro tema apresentou a jornada dos pais para acessar um diagnóstico adequado para o filho, algumas vezes os sintomas apresentados pelo paciente eram confundidos pelos profissionais de saúde com outros problemas de saúde, atrasando em anos alguns diagnósticos. A assistência à saúde brasileira é notoriamente heterogênea e muitas regiões do país ainda carecem de formação profissional relacionada à oncologia pediátrica e infraestrutura adequada, o que dificulta a detecção do câncer precoce na infância. Os serviços especializados de atenção ao câncer estão mais concentrados nas grandes cidades e nas regiões mais desenvolvidas dos países, o que também contribui para a desigualdade no tratamento do câncer infantojuvenil^34^. Assim, estas diferenças regionais na prestação destes serviços fazem com que as taxas de sobrevivência no país ainda estejam aquém dos esperados para o desenvolvimento e conhecimento técnico do momento atual. Isso acontece porque questões inerentes à organização do sistema de saúde e ao acesso oportuno ao diagnóstico contribuem para determinar diferentes chances de sobrevivência^34^ ^)-(^ ^35^.

Os resultados mostraram que os pais e os seus filhos enfrentam desafios multifacetados no diagnóstico, tratamento e na sobrevivência ao câncer infantojuvenil. A experiência vivida marca profundamente as suas vidas, de forma que integram uma nova identidade após o câncer, e que não é possível ser o mesmo que era antes da doença. Nove dos dez pais participantes descreveram seus filhos como sobreviventes de câncer infantojuvenil, e também atribuíram a eles e às famílias a identidade de “sobrevivente”. A definição de sobrevivente de câncer do Instituto Nacional do Câncer (INCA) inclui familiares, amigos e cuidadores, pois também são afetados pelo câncer^10^ ^),(^ ^36^. Porém, um dos pais deste estudo não identificou seu filho e sua família como sobreviventes e da mesma forma, a literatura mostra que de fato nem todos se identificam com o termo sobrevivente^37^ ^)-(^ ^38^. As evidências indicam que a identificação de não ser um sobrevivente do câncer é muitas vezes influenciada pelo curso do tratamento (por exemplo, não requer quimioterapia e radiação), um prognóstico favorável, bem como pela confiança nos prestadores de cuidados oncológicos^39^ ^)-(^ ^40^.

Viver após o câncer é uma experiência persistentemente “disruptiva”^41^ ^)^ e mesmo na sobrevivência a longo prazo, os níveis de estresse parental desencadeados pelo diagnóstico e tratamento podem não diminuir^42^ ^)-(^ ^43^. Os resultados deste estudo mostraram que o medo vivenciado pelos pais afeta a sua paz na sobrevivência e também a sua qualidade de vida. As evidências mostram níveis elevados de sofrimento psicológico, como sintomas de estresse pós-traumático, depressão, ansiedade, distúrbios do sono, sintomas somáticos, medo de recidiva, preocupação extensa e fadiga entre pais de sobreviventes de câncer infantil^42^ ^)-(^ ^44^. A incerteza após o tratamento e a necessidade de lidar com o desconhecido levam os pais a um ciclo de hipervigilância e, portanto, ao sofrimento contínuo^45^. Em consonância com os resultados deste estudo, a literatura mostra que as mães são os familiares que mais se sentem afetados pelo sofrimento psíquico^46^ ^)-(^ ^47^.

Apesar disso, as narrativas descrevem que os pais acreditavam que, após o “renascimento”, eles se percebiam como pessoas melhores, assim como seus filhos, com novas expectativas de vida. Resultados positivos também podem ocorrer após um evento estressante, como o diagnóstico de câncer infantojuvenil^48^, processo definido na literatura como crescimento pós-traumático^48^ ^)-(^ ^49^. O crescimento pós-traumático envolve os processos de construção de novos significados após um evento traumático, que pode incluir a descoberta de benefícios e o desenvolvimento pessoal^48^ ^)-(^ ^49^.

Este estudo apresenta pontos fortes, que incluem o método narrativo, descrição rigorosa do recrutamento dos participantes, entrevistas semiestruturadas, abrangentes e densas, análise rigorosa dos dados com dois codificadores independentes e construção de temas e subtemas com toda a equipe de pesquisa. Além disso, os resultados apresentados neste estudo esclarecem a experiência de ser pai de um sobrevivente de câncer infantojuvenil e chamam a atenção para a necessidade de estudos futuros, e que os profissionais de saúde e a mídia precisam estar sensíveis a essa experiência, que ainda é pouco compreendida na literatura. No entanto, as limitações do estudo são reconhecidas. A realização de uma única entrevista com os pais pode ter limitado a profundidade de compreensão de aspectos importantes da experiência descrita pelos participantes na primeira entrevista. Foram incluídos pais em duas fases distintas da sobrevivência ao câncer infantojuvenil, estendida e permanente, o que pode ter limitado a compreensão das singularidades da experiência de ser pai em cada um desses estágios de sobrevivência. Além disso, embora em outros estudos que incluem os pais a representação das mães seja superior à dos pais, a inclusão de apenas um pai na amostra deste estudo também deve ser reconhecida como uma limitação.

Este estudo abre caminho para pesquisas futuras com pais de sobreviventes de câncer infantil. É importante que estudos futuros explorem a experiência com estruturas familiares e contextos culturais diferentes dos participantes incluídos neste estudo, a fim de discutir melhor as narrativas dos pais aqui descritas e acrescentar novas percepções sobre a experiência vivida por eles durante a sobrevivência. Embora o estudo tenha sido desenvolvido durante a pandemia de COVID-19, não foi nosso objetivo explorar a influência da pandemia de COVID-19 na experiência destes pais. Futuros estudos qualitativos podem explorar a influência da pandemia de COVID-19 na experiência dos pais de sobreviventes de câncer infantojuvenil. Além disso, a investigação futura deverá desenvolver modelos de cuidados de sobrevivência centrados na família, com o objetivo de minimizar o impacto do câncer nas suas vidas. O atual modelo de cuidados à sobrevivência ao câncer em crianças e adolescentes ainda não inclui a família, o foco está no paciente. Na área da educação, a perspectiva apresentada nos resultados deste estudo pode orientar o ensino de graduação e pós-graduação, capacitando profissionais de saúde para serem mais sensíveis às particularidades das experiências dos pais dos sobreviventes do câncer infantojuvenil. Pesquisas futuras também podem procurar possíveis diferenças nas experiências entre pais e mães de sobreviventes de câncer infantojuvenil. Este é um dado interessante para que os enfermeiros possam adequar os seus cuidados, levando em conta também a questão de gênero nos cuidados de enfermagem além do câncer.

Por fim, os resultados deste estudo trazem uma série de implicações clínicas para o escopo da enfermagem, independentemente do cenário de atuação, atenção especializada ou básica. Primeiramente, é necessário que os gestores de saúde invistam na qualificação profissional para possibilitar o reconhecimento oportuno dos sinais e sintomas do câncer infantojuvenil e, posteriormente, reduzir o tempo de diagnóstico. O enfermeiro pode atuar na identificação de sinais e sintomas do câncer em crianças e adolescentes, contribuindo para um acesso mais rápido ao atendimento médico. Conforme discutido anteriormente, o diagnóstico oportuno aumenta as taxas de sobrevivência ao câncer infantojuvenil. Neste estudo, os pais utilizaram metáforas para relatar suas experiências em relação à sobrevivência de seus filhos ao câncer infantojuvenil. As metáforas têm potencial para contribuir para um cuidado de enfermagem qualificado, centrado no paciente e na família, pois ajudam a traduzir a experiência da doença e a tornam acessível ao sobrevivente e aos seus pais^50^. O sistema de metáforas também pode contribuir para a criação de outros significados e conceitos relacionados ao câncer, que ainda é estigmatizado e visto como diagnóstico de morte. Porém, deve ser utilizado com respeito e sensibilidade, observando a forma de comunicação escolhida pela família e paciente. Os resultados também mostraram que o medo da recorrência tem impacto na paz dos pais, incluindo na qualidade de vida dos pais. Os profissionais de saúde podem considerar a importância de oferecer espaços para os pais expressarem seus medos e sentimentos, bem como informações relacionadas à recidiva do câncer infantojuvenil, mitigando assim o impacto desse medo em suas vidas. Infelizmente, grande parte dos cuidados de sobrevivência ao câncer infantojuvenil concentram-se no atendimento clínico, sem dar atenção à importância da abordagem psicoemocional.

## Conclusão

Os resultados desses estudos trazem uma nova perspectiva que mostra que a experiência dos pais durante a sobrevivência ao câncer infantil é permeada por tempos de guerra e tempos de paz incerta. Os resultados desta pesquisa têm o potencial de fornecer uma compreensão mais profunda e coerente das experiências dos pais dos sobreviventes de câncer infantojuvenil na sobrevivência estendida e permanente, bem como ajudar a delinear melhores cuidados de enfermagem baseados em evidências para eles. Além disso, este conhecimento pode ser utilizado pelos enfermeiros para desenvolver modelos de cuidados centrados na família para os sobreviventes e melhorar, a longo prazo, a qualidade de vida dos pais, bem como dos próprios sobreviventes.

# Translation: “Tiempos de guerra y tiempos de paz incierta”: narrativas de padres de supervivientes de cáncer infantojuvenil*

**DOI:** 10.1590/1518-8345.7005.4262

**Authors:** Rhyquelle Rhibna Neris, Ana Carolina Andrade Biaggi Leite, Elizabeth Papathanassoglou, Cristina Garcia-Vivar, Juliana de Souza, Lucila Castanheira Nascimento

**Affiliations:** 1 Universidade de São Paulo, Escola de Enfermagem de Ribeirão Preto, Centro Colaborador de la OPS/OMS para el Desarrollo de la Investigación en Enfermería, Ribeirão Preto, SP, Brasil.; 3 Universidad Pública de Navarra, Facultad de Ciencias de la Salud, Pamplona, Navarra, España.; 4 University of Alberta, Faculty of Nursing, Edmonton, Alberta, Canadá.; 5 Neurosciences, Rehabilitation & Vision Strategic Clinic Network, Alberta Health Service, Edmonton, Alberta, Canadá.

**Keywords:** Sobrevivientes, Neoplasias, Niño, Adolescente, Padres, Investigación Cualitativa

## Abstract

**Objetivo:**

analizar los significados que les atribuyen los padres a la supervivencia prolongada y permanente del cáncer infantojuvenil.

**Método:**

estudio narrativo cualitativo, desarrollado con padres de adolescentes y adultos jóvenes que sobrevivieron al cáncer infantojuvenil. El reclutamiento y la recogida de datos se basó en enfoques virtuales y presenciales. Los datos fueron recolectados a través de entrevistas semiestructuradas y analizados según análisis temático reflexivo.

**Resultados:**

se incluyeron en el estudio un total de diez padres. Se construyeron dos síntesis narrativas temáticas: “Tiempos de guerra”; y “Tiempo de paz incierta”, con sus respectivos subtemas. El diagnóstico de cáncer marca el inicio de tiempos de guerra en la vida de los padres. Viven el tratamiento del cáncer como “altibajos” con amenazas potenciales para la vida de sus hijos. Después llega el “Tiempo de paz incierta” y se restablece el equilibrio de la unidad familiar. Pero el miedo a la recidiva torna incierta la paz familiar, y mantenerla implica un control constante y prestar atención a los signos y síntomas de una posible nueva batalla.

**Conclusión:**

los resultados destacan la experiencia de ser padre de un superviviente de cáncer infantil y pueden aplicarse para desarrollar modelos de atención centrados en la familia de los supervivientes.

## Destacados

**(1)** La literatura aún no incluye las experiencias de los padres de los niños que sobrevivieron al cáncer.

**(2)** El tratamiento agudo del cáncer infantil se vive como una época de guerra.

**(3)** Los padres viven la supervivencia a largo plazo como un momento de paz.

**(4)** El miedo a la recidiva hace incierta la paz de los padres, incluso en la supervivencia a largo plazo.

**(5)** Hay que incluir a los padres en el cuidado de los supervivientes.

## Introducción

El cáncer infantojuvenil a menudo se define como el cáncer que ocurre entre el nacimiento y los 14 años de edad, pero también puede incluir cánceres hasta los 18 o 19 años de edad^1^ ^)-(^ ^3^. Las opciones de tratamiento para el cáncer infantojuvenil han mejorado en las últimas décadas. A pesar de los avances, hay que considerar que la supervivencia mundial después de cinco años varía considerablemente según la región, es del 8,1% en África Oriental, del 73% en Europa, del 60% en América del Sur y llega hasta el 83,0% en América del Norte^4^. A nivel mundial, la tasa de supervivencia estimada es del 37,4% durante los mismos 5 años^4^. Dicha variación destaca las desigualdades en salud entre diferentes países^5^. Los supervivientes de leucemia representan aproximadamente un tercio de todos los supervivientes de cáncer menores de 20 años^6^.

A lo largo de los años han surgido nuevas definiciones de lo que constituye un supervivientes de cáncer^7^ ^)-(^ ^8^. Los supervivientes han sido definido de diversas formas como aquellos a quienes se les diagnosticó cáncer por primera vez o aquellos que han vivido con un diagnóstico de cáncer durante 5 años o más^7^. La primera definición del concepto de supervivientes de cáncer la creó Mullan en 1985^9^, médico y supervivientes de cáncer, quien presentó las tres “estaciones de supervivencia”^9^, que son: supervivencia aguda (período posterior al diagnóstico, donde la atención se centra en el tratamiento); supervivencia prolongada (período posterior al final del tratamiento, a menudo conocido como remisión, donde la atención se centra en afrontar las consecuencias a corto plazo del tratamiento); y supervivencia permanente (5 años después del final del tratamiento, cuando el paciente está “curado” pero debe hacer frente a los efectos tardíos del cáncer y los tratamientos). Esta es la definición que adoptamos en este estudio^9^.

La familia también es considerada superviviente del cáncer infantojuvenil^7^ ^)-(^ ^8^ ^),(^ ^10^, y muchas veces se la denomina “superviviente secundaria”. Aunque el cáncer no afecta biológicamente a los padres, definitivamente, sí los afectada funcional, social y emocionalmente. Los padres pueden experimentar una variedad de efectos después del tratamiento de sus hijos, sufrimiento psicológico y trastorno de estrés postraumático, pérdida de ingresos y toxicidad financiera debido a la elevada carga de gastos experimentada durante, e incluso, después del tratamiento del cáncer, miedo a la recidiva de la enfermedad y la necesidad de abordar los efectos físicos tardíos que enfrentan los niños después del tratamiento^11^ ^)-(^ ^13^. Los estudios demuestran que dichos efectos contribuyen a la mala calidad de vida de los padres^14^.

Sin embargo, en la literatura aún no se incluye la experiencia de los padres y es poco lo que se sabe sobre la experiencia que tienen durante la supervivencia del cáncer infantojuvenil. La literatura hasta la fecha incluye principalmente estudios realizados durante el tratamiento activo, que se centran en las experiencias durante la quimioterapia^15^ ^)-(^ ^16^, la etapa final de la vida o el duelo de los padres^17^ ^)-(^ ^18^, desde la perspectiva única del superviviente de cáncer^19^ ^)-(^ ^21^, o desde la perspectiva de los cuidadores familiares de adultos supervivientes^22^. Por lo tanto, este estudio tuvo como objetivo responder a este vacío de conocimiento y aportar las perspectivas de los padres de supervivientes de cáncer infantojuvenil.

La pregunta de investigación que orientó este estudio fue: ¿Cómo viven los padres de supervivientes de cáncer infantojuvenil en las fases de supervivencia prolongada y permanente? Este estudio cualitativo tuvo como objetivo analizar los significados que les atribuyen los padres a la supervivencia prolongada y permanente del cáncer infantojuvenil.

## Método

### Diseño

Se trata de una investigación narrativa cualitativa, basada en el enfoque metodológico de Squire^23^ de la narrativa centrada en la experiencia. Las narrativas construidas a partir del modelo centrado en la experiencia involucran movimiento, sucesión, progresión o secuencia, son generalmente temporales e implican la articulación o el desarrollo del significado de la experiencia que vive el narrador^23^. Una narrativa debe contener cinco elementos esenciales: trama (conjunto de hechos); personajes (quién realiza la acción); tiempo (duración y momento en el que se desarrolla la historia); espacio (lugar donde ocurre la acción) y entorno (espacio atravesado por las características socioeconómicas, morales y psicológicas en el que viven los personajes)^24^.

### Ámbito de estudio

Consulta externa de oncohematología pediátrica de un hospital pediátrico ubicado en el interior del estado de São Paulo (Brasil). Esta clínica ambulatoria brinda servicios de atención a supervivientes de cáncer infantojuvenil y pacientes con enfermedades hematológicas benignas.

### Participantes y criterios de elegibilidad

Utilizamos un muestreo intencional para invitar a padres de adolescentes o adultos jóvenes supervivientes de cáncer infantojuvenil. Los criterios de elegibilidad incluyeron: ser mayor de 18 años; ser padre de un adolescente o adulto joven con cáncer y haber participado en su cuidado. Se define adolescente y adulto a cualquier individuo entre 15 y 39 años. Todos los hijos supervivientes de los participantes en este estudio completaron el tratamiento contra el cáncer infantil y se encontraban en las etapas de supervivencia prolongada y permanente del cáncer infantil.

El reclutamiento y la recogida de datos para esta investigación se realizó entre mayo de 2021 y agosto de 2022. Debido a las restricciones sociales provocadas por la pandemia de COVID 2019, el reclutamiento de los participantes se realizó de forma híbrida, mediante reclutamiento presencial y remoto, como se detalla a continuación:

- Reclutamiento presencial: realizado en el servicio ambulatorio de oncohematología pediátrica. El investigador principal identificó a los padres elegibles a través de la lista de consultas médicas. Se contactó a los potenciales participantes el día de la cita médica del adolescente o adulto joven supervivientes. Los padres fueron invitados a participar y recibieron información sobre los procedimientos del estudio. Quienes aceptaron participar firmaron un formulario de consentimiento libre e informado por escrito. Posteriormente, se programó la entrevista con el participante, según la modalidad que eligiera: 1) presencial, en el servicio ambulatorio, mientras esperaba a la consulta médica; o 2) *online* en la plataforma que eligiera y con la que se sintiera más familiarizado (*Google Meet*, *Zoom* o *WhatsApp* vídeo);
- Reclutamiento remoto: realizado *online*, a través de plataformas como *Instagram* y *WhatsApp*, y por teléfono. Cuando el responsable estaba ausente en el momento del encuentro presencial en el servicio ambulatorio, el investigador se acercaba al supervivientes el día de la consulta médica, antes o después de la consulta, y le explicaba los objetivos y procedimientos de la investigación, y cuando el supervivientes daba su consentimiento, se le solicitaba el número de teléfono de los padres. Posteriormente, se contactó con los padres y se les dio una explicación detallada del objetivo y los procedimientos del estudio, y de cómo la investigadora obtuvo su contacto telefónico. Aquellos que expresaron interés en participar en el estudio recibieron un *link* para completar el formulario de consentimiento en *Google Forms*. Además, también se invitó a los padres a participar en el estudio a través de *Instagram*. Los participantes reclutados en *Instagram* no realizaban el seguimiento en la clínica ambulatoria de oncohematología pediátrica, que era el principal lugar de reclutamiento. A través de un perfil social que publica contenido relacionado con el cáncer y también informes de experiencias públicas de los supervivientes, se identificaron supervivientes de cáncer infantojuvenil. Mediante mensajes directos de *Instagram*, la investigadora les envió una invitación a los posibles participantes, que contenía información sobre los objetivos y procedimientos de la investigación; en ese momento también se respondieron a preguntas. Después de que manifestaron que querían participar en el estudio, se les envió el *link* para completar el formulario de consentimiento libre e informado.

### Recogida de datos

La recogida de datos fue realizada por una estudiante de doctorado especialista en enfermería oncológica (primera autora del estudio), con experiencia previa en la recogida de datos cualitativos, y que no tenía conexión previa con los participantes de la investigación. Los datos del estudio se recolectaron a través de entrevistas individuales *online*, mediante videollamadas de *WhatsApp* y *Google Meet*; y entrevistas individuales presenciales, realizadas en una sala privada del servicio ambulatorio del hospital. Se realizaron ocho entrevistas *online* y dos entrevistas presenciales, una a cada participante. En ambas modalidades se realizaron entrevistas semiestructuradas, utilizando una guía de entrevista preestablecida, elaborada por las investigadoras, que tenía preguntas orientadoras, como: “¿Puedes contar la historia desde los primeros signos y síntomas de cáncer de tu hijo?”, “¿Puedes contarnos la historia sobre el tratamiento contra el cáncer de tu hijo?”, “¿Alguna vez has oído hablar de la supervivencia al cáncer?”, “¿Qué opinas de esta expresión?”. Además, se introdujeron preguntas de seguimiento, como: “¿Podrías contarme más sobre esto?” Estas preguntas permitían que la historia se desarrollara como el participante decidía contarla. Además, los autores utilizaron un formulario para recopilar datos relacionados con la caracterización de los padres y supervivientes de cáncer infantil.

Tanto las entrevistas presenciales como las *online* fueron grabadas con una grabadora de voz externa, que no captaba las imágenes de los participantes. Se realizó la transcripción completa de los audios de las entrevistas para realizar el análisis de datos. No se les enviaron las transcripciones a los participantes. Sin embargo, un investigador trascribió las entrevistas y un segundo investigador llevó a cabo una revisión completa para verificar su veracidad y corregir posibles errores. El análisis se realizó simultáneamente con la recogida de datos. Esta técnica permitió finalizar la recogida cuando los datos de las entrevistas comenzaron a repetirse y no se encontraba ningún significado o código nuevo, ocurriendo ésto en la entrevista número 10^25^ ^)-(^ ^26^.

### Tratamiento y análisis de datos

El método narrativo permite el uso de diferentes tipos de métodos de análisis de datos y el más común es el análisis temático^27^ ^-^ ^28^. Por lo tanto, para este estudio se utilizó el método de análisis temático reflexivo^29^. El proceso se describe en la Figura 1.

**Figura 1:** Estructura del análisis de dados. Ribeirão Preto, SP, Brasil, 2022

Estadio I	Las entrevistas fueron transcritas en su totalidad. Posteriormente, para familiarizarse con las entrevistas, dos investigadores leyeron activamente todas las entrevistas en busca de patrones y significados, y anotaron las ideas principales para la codificación.
Estadio II	La codificación línea por línea de las entrevistas fue realizada de forma independiente por dos autores (primera y penúltima autora), mediante el *software* MAXQDA2020^®(^ ^30^. En esta etapa se generaron en total 180 códigos *in vivo*. Los códigos generados identificaron aspectos latentes de los datos y las características que dieron forma y significados específicos a los datos. Después de la codificación independiente inicial, se llevó a cabo una discusión entre todos los autores y el cruzamiento de todos los códigos obtenidos. En esta etapa, la codificación se basó en agrupar los datos recolectados para luego identificar la trama de las narrativas.
Estadio III	Una vez codificados todos los datos, considerando las relaciones y diferencias entre los códigos identificados en la etapa anterior, se construyeron de forma inductiva dos síntesis narrativas. La principal característica de una síntesis narrativa es integrar la experiencia de todos los participantes, considerando sus convergencias y divergencias. Además, mantiene la esencia de las narrativas de los participantes para ejemplificar estas experiencias. Este paso lo realizaron el primer y último autor del estudio.
Estadio IV	En la siguiente fase, a partir de las narrativas, el primer y el último autor identificaron y nombraron de forma inductiva los dos temas interpretativos.
Estadio V	Finalmente, en la última fase del análisis temático reflexivo, se refinaron los temas previamente construidos y se definieron los temas de las narrativas finales del estudio, así como los subtemas que los estructuran. Este paso se completó mediante la discusión del equipo de investigación y la revisión cuidadosa del informe de resultados. La quinta etapa del análisis también garantizó la coherencia de los datos presentes en los temas.

### Consideraciones éticas

El estudio fue aprobado por los comités de ética de la institución proponente y coparticipante, con dictamen N.º 012/2020, del 24/01/2020. Los participantes recibieron toda la información relacionada con la investigación antes de comenzar el estudio. Se obtuvo el consentimiento informado de todos los participantes.

### Rigor metodológico

Las siguientes consideraciones aumentan el rigor metodológico de este estudio^31^ ^)-(^ ^32^: 1) Credibilidad: el análisis de los datos fue realizado por un equipo de investigación, mediante la presentación de códigos, temas y subtemas identificados y la presentación de citas relevantes de las narrativas. Además, se presentan extractos de las narrativas para que el lector pueda ver que las narrativas realmente presentan los significados que los participantes les dan a sus experiencias; 2) Transferibilidad: presentación metodológica, de forma transparente, de los procesos metodológicos y analíticos de toma de decisiones de la investigación y descripción del contexto sociocultural de los participantes; 3) Fiabilidad: descripción rigurosa de los aspectos metodológicos de acuerdo con el *Consolidated Criteria for Reporting Qualitative Studies* - COREQ^33^ y del tiempo adecuado dedicado a la interacción con los participantes; y 4) Confirmabilidad: presentación de las limitaciones y puntos fuertes del estudio y las reflexiones de los investigadores durante el proceso de investigación.

## Resultados

Se invitó a quince padres a participar en el estudio, tres de los cuales rechazaron la invitación porque no estaban disponibles para participar. Dos padres aceptaron la invitación y dieron su consentimiento para participar en la investigación, pero posteriormente no respondieron a los contactos para programar la entrevista *online*. Después de tres intentos fallidos de programar la entrevista, fueron excluidos del estudio. Al final, participaron en el estudio diez padres de niños y adolescentes supervivientes de cáncer, nueve eran madres y uno padre, y todos acompañaron el proceso de diagnóstico y tratamiento del paciente. Las entrevistas tuvieron una duración promedio de 75 minutos, el tiempo mínimo fue de 31 minutos y el máximo de 136 minutos.

La edad de los participantes osciló entre 37 y 56 años. Considerando que los participantes reclutados e incluidos en el estudio a través de la muestra por conveniencia eran predominantemente madres, se buscó incluir a un padre para comprender la perspectiva de género e identificar posibles variaciones en la experiencia de supervivencia según el género. Sólo uno de los padres estuvo disponible durante el proceso de reclutamiento. La caracterización de los participantes se presenta detalladamente en la Tabla 1.

**Tabla 1:** Caracterización de los padres y supervivientes de cáncer infantojuvenil. Ribeirão Preto, SP, Brasil, 2022

Características de los participantes	*n*
**Relación con el adolescente o adulto joven**	
Madre	9
Padre	1
**Edad (años)**	
31 - 40	3
41 - 50	6
51 - 60	1
**Género**	
Femenino	9
Masculino	1
**Estado civil**	
Soltero	1
Casado	7
Unión estable	2
**Educación**	
Enseñanza primaria	4
Enseñanza media	3
Educación superior	2
Características de los supervivientes	*n*
**Edad (años)**	
11 - 15	3
16 - 20	5
21 - 25	1
26 - 30	1
**Género**	
Femenino	4
Masculino	6
**Diagnóstico del supervivientes**	
Leucemia	3
Tumor del Sistema Nervioso Central	3
Sarcoma de Tejido Blando	1
Osteosarcoma	1
Tumor de Wilms	1
Linfomas	1
**Tiempo desde el diagnóstico**	
2 años	2
3 años	1
4 años	4
5 años o más	3
**Tratamientos**	
Quimioterapia (QT)	4
Cirugía	1
Quimioterapia + Radioterapia	1
Quimioterapia + Cirugía	2
Quimioterapia + Radioterapia + Cirugía	1
Radioterapia + Cirugía	1

La presentación de los resultados se estructura en dos narrativas principales: (1) “Tiempos de guerra”; y (2) “Tiempos de paz incierta”, cada uno con sus subtemas, que se ilustran en la Figura 2. Todos los temas, subtemas y narrativas adicionales de los participantes se presentan en la Figura 3.

**Figura 2:** Síntesis de los temas y subtemas

**Figura 3:** Temas, subtemas y narrativas seleccionadas. Ribeirão Preto, SP, Brasil, 2022

“Tiempos de guerra”	**Subtemas**	**Narrativas de los participantes**
	“*Un niño cuya vida se detuvo a los 9 años*”	*Cuando tenía unos 5 años, empezó a tener dolor de cabeza y vómitos. Esto duró varios meses, tenía vómitos y dolor de cabeza, lo llevaba al hospital, le hacían exámenes y no encontraban nada. Con el tiempo comenzaron a aparecer otros síntomas, le costaba mucho ver y hablar, tenía la voz muy ahogada, muy arrastrada, había momentos que ni siquiera entendíamos lo que decía* (Madre, p2).
		*En una ocasión tuvo una convulsión muy fuerte, lo llevamos al hospital, los médicos y profesionales de la salud dijeron que estaba drogado. Le dije: “No, mi hijo no consume drogas. Es un niño cuya vida se detuvo a los nueve años. No consume drogas”. Ese fue el peor día de mi vida* (Madre, p7).
	Confirmación del diagnóstico de cáncer: impacto en la familia	*No tenía ninguna esperanza. Sinceramente, en ese momento pensé: “Creo que me voy a subir al auto, salir a la carretera y matarme con mi hijo”. Creo que me voy a matar, no sólo yo, sino también a mi hijo. No voy a aguantar esto* (Madre, p1).
		*Cuando mi hija fue diagnosticada, tenía anemia grave debido a la leucemia. Ella corría un gran riesgo, tenía un 90% de posibilidades de morir, un 10% de posibilidades de sobrevivir* (Padre, p4).
	“*Altibajos*” durante el tratamiento	*Para poder seguir el tratamiento, ella y yo nos mudamos a otra ciudad y prácticamente vivimos en el hospital. No vi a mi hijo, no vi a mi marido. No había nadie que me pudiera remplazar y pasar un poco de tiempo con ella en el hospital, para poder salir del hospital. No había nadie. Fue un período muy, muy desafiante para mí, porque pensé que no iba a poder. No fue fácil. Realmente. El tratamiento fue muy difícil. Pensé que me iba a volver loca* (Madre, p3).
		*Pasamos por momentos difíciles. Menstruó durante el tratamiento y sufrió una hemorragia. El flujo era abundante, estaba desesperado. Tuvo líquido en los pulmones debido al ATRA (ácido transretinoico) y su saturación bajó al 80%. Esa vez fue trasladada a la UCI (Unidad de Cuidados Intensivos). Fue un momento muy difícil, pero ella lo superó. Hubo un momento en que contrajo citomegalovirus y estuvo hospitalizada durante 40 días. Pasó 25 días internada sin que se supiera qué era. Pensé que se iba a morir, que no lo descubrirían, pensamos que había vuelto la leucemia. Hubo muchos altibajos en el tratamiento* (Padre, p4).
“Tiempos de paz incierta”	*“Mi hijo es un* superviviente *, es un guerrero”*	*¡Hay tiempos de guerra y hay tiempos de paz! Ahora ha llegado el momento de que todos levantemos la bandera blanca de la paz. Hoy en día solo recordamos lo que pasamos y le pedimos a Dios que no vuelva a pasar por eso bajo ninguna circunstancia, que Dios nos ayude para que esto no suceda. Ahora somos una familia bien estructurada y equilibrada. Ahora ya todo pasó. Por eso digo que hoy es tiempo de paz, el tiempo de guerra termin*ó*. Hoy somos vencedores, por todo lo que pasó; hoy somos vencedores* (Madre, p7).
		*Él es un superviviente del cáncer. Sobrevivió al no entrar en coma, sobrevivió a la primera cirugía, a la segunda cirugía y también a la quimioterapia. Sobrevivió a todo eso. Sobrevivió tres veces. ¡Es un superviviente muy superviviente!* (Madre, p2).
	*“Es un renacimiento”*	*Hoy puedo ver que ella es una ganadora. Lo digo así: “dejaste la muerte para renacer de nuevo, es un renacer, un resurgimiento”. Eso es la supervivencia, sobrevives en la fase crítica y sobrevives y eres una mejor persona después* (Padre, p4).
		*El hospital fue extraordinario en la vida de mi hijo y lo trajo de vuelta. Hoy está feliz, lo superó todo* (Madre, p7).
		*Mi hija nació de nuevo. Dios la sanó y no murió. Ella revivió de nuevo* (Madre, p9).
	Alianzas para lograr tiempos de paz	*Para ser un* superviviente*, necesita que la familia no abandone el tratamiento. Si no hubiera tenido mi apoyo, el de su padre y de otros familiares y amigos,* él *se hubiera rendido* (Madre, p3).
		*Toda mi familia sobrevivió al cáncer. Toda la familia es superviviente, la familia está unida y la familia son vínculos. Cuando un miembro pasa por algo, la familia pasa por eso unida. Por todo lo que hemos pasado, obviamente todos somos* supervivientes *del cáncer* (Madre, p7).
	*“La madre de un* supervivientes *nunca se siente aliviada”*	*Da mucho miedo. Mucho miedo. Cada vez que vamos a un examen sentimos ese miedo. Da miedo llegar al hospital y decir algo. Por mucho que tengamos fe en Dios y creamos que él no volverá, es algo que me asusta mucho. No sé él, además prefiero no meterle nada en la cabeza para que no tenga que preocuparse por eso, por algo que ni siquiera está pasando. Déjamelo a mí, soy madre* (Madre, p3).
		*Aunque ella está bien, todavía hay esa inseguridad dando vueltas. Todavía no me siento segura. Esto afecta un poco mi calidad de vida; no duermo y pienso. A veces estoy callada y pensativa; de repente vienen los recuerdos y eso me asusta* (Madre, p5).

Las síntesis narrativas muestran que el diagnóstico de cáncer en niños y adolescentes marca el inicio de tiempos de guerra en la vida de los padres y provocan un desequilibrio en el núcleo familiar. Como en toda guerra, los padres narraron que el tratamiento del cáncer estaba lleno de “altibajos”, y presentaba varias amenazas potenciales para la vida de sus hijos. Después de los “Tiempos de Guerra”, viene un período de “Tiempos de paz incierta”. En esta etapa se restablece el equilibrio de la unidad familiar y se inicia la fase de supervivencia al cáncer infantil. Aunque la guerra ha terminado, los padres describieron que el miedo a la recidiva es un factor que perturba constantemente la paz familiar, volviéndola incierta. Por ende, describieron la paz como precaria e incierta, dado que mantenerla requiere el control constante de los padres, que siempre están atentos a los signos y síntomas de una posible nueva guerra.

### 1. Tema: “Tiempos de Guerra”

1. Subtema: *“Un niño cuya vida se detuvo a los 9 años”*: Los tiempos de guerra interrumpen la vida de los afectados. Además, los padres informaron que la vida de sus hijos se vio interrumpida durante el período anterior al diagnóstico de cáncer. Los padres mencionan una serie de síntomas físicos que no son normales y los alertan sobre la necesidad de buscar respuestas y tratamiento para sus hijos. La fase de prediagnóstico está marcada por idas y vueltas a las instituciones de salud, que en algunos casos duran varios meses e incluso años.

Una madre describió que la epilepsia sintomática que venía padeciendo su hijo, debido al tumor cerebral, fue interpretada durante algún tiempo como un trastorno psiquiátrico y abuso de drogas por los profesionales de la salud. Los padres utilizan la analogía de “tiempos de guerra” para describir la experiencia que tuvieron durante la búsqueda del diagnóstico, debido a todos los cambios en la vida cotidiana del niño y de toda la familia, que provocó un desequilibrio en el núcleo familiar: *Le tomó un tiempo recibir el diagnóstico, comenzó a tener convulsiones cuando tenía 9 años. Tenía 9, 10, 11, 12, 13, 14, 15... iba a cumplir 16 años. Pasaron muchos años, muchos, muchos años hasta que le dieron el diagnóstico. Mi vida se detuvo desde el momento en que empezó a convulsionar. Fue un golpe para la familia, un desequilibrio para toda la familia. ¡¡Vaya, eran como tiempos de guerra!! (Madre, p7)*

Estar en guerra requiere detener la vida y centrarse en un objetivo: “ganar la guerra”. Para ello, los padres narraron cómo cambió su vida desde que aparecieron las primeras señales de cáncer.

2. Subtema: Confirmación del diagnóstico de cáncer: impacto en la familia: Después del largo camino para llegar al diagnóstico, la confirmación del cáncer se vive como un momento “horrible”, de “desesperación”, sienten “que les falta la tierra bajo los pies” y un “cimbronazo”. El estigma del cáncer hace que los padres piensen que es un diagnóstico mortal. *Mi hijo se va a morir, eso es lo que tienes en mente... Hoy es muy difícil ver que sobreviví al cáncer porque es muy agresivo y nos asusta a todos. Viví un día a la vez. ¡¡Fue horrible!! Me aterraba escucharlos decir el nombre de mi hijo por el altavoz para entrar al consultorio. Realmente estaba en un desierto” (Madre, p1).*

El diagnóstico de cáncer del hijo despertó el sentimiento de “querer morir” e ideas suicidas, debido a la desesperación de la noticia y la falta de apoyo que sentía en ese momento. Las narrativas muestran que los padres entraron en un ciclo de desesperanza que les impedía creer que sus hijos responderían bien al tratamiento oncológico que estaban por comenzar y que tendrían un buen pronóstico.

3. Subtema: “Altibajos” durante el tratamiento del cáncer: El período de tratamiento estuvo marcado por la dificultad de acceso a los centros oncológicos para el tratamiento del cáncer infantojuvenil y por la separación geográfica de la unidad familiar, necesaria para que el niño enfermo pueda seguir el tratamiento. Para algunos de los entrevistados, las madres asumieron el papel de cuidadoras de sus hijos enfermos y los padres cuidaron de sus hermanos sanos. Además, las narrativas muestran que muchos padres tuvieron que dejar sus trabajos para poder acompañar a su hijo a las internaciones y atender las demandas del tratamiento.

Como toda guerra llena de batallas, el tratamiento fue descripto por los padres como plagado de “altibajos”. Para “combatir el cáncer”, los niños y adolescentes se sometieron a distintos tipos de tratamientos, como: quimioterapia, cirugía, inmunoterapia y radioterapia. Los padres describieron varios de los efectos adversos de estos tratamientos que culminaron en complicaciones graves y potencialmente mortales, que incluso requirieron ingreso a la unidad de cuidados intensivos. La posibilidad de estar vivo al final del proceso de tratamiento y ser superviviente se vio amenazada en varios momentos durante los tiempos de guerra.

### 2. Tema: “Tiempos de paz incierta”

1. Subtema: *“Mi hijo es un* superviviente*, es un guerrero”*: Después de los “tiempos de guerra”, en los que el proceso de diagnóstico y tratamiento oncológico se vivió con altibajos y potenciales amenazas para la vida del hijo, las narrativas muestran que llegaron los “tiempos de paz incierta”, la fase de sobrevivir al cáncer. En tiempos de paz, el equilibrio familiar se restablece y los momentos difíciles vividos en tiempos de guerra se convierten en recuerdos que marcarán para siempre la vida de los padres. Casi por unanimidad, nueve participantes del estudio se refirieron a sus hijos como supervivientes del cáncer. Sólo una madre dijo que no consideraba que su hijo era un superviviente, ya que el término hacía referencia a algo negativo y prefería usar el término “curado” para referirse a su hija. *Mi hijo ha pasado por mucho, ¡mi hijo es un superviviente! Es un guerrero, a pesar de toda la experiencia por la que pasó y sobrevivió. Luchó mucho por sobrevivir y tuvo mucha determinación. Cuando terminó la quimioterapia y le dieron el alta para volver a casa, les dije a las enfermeras: “Mi hijo es un superviviente, es un guerrero”. ¡Sobrevivió a todo! (Madre, p6)*

Los participantes utilizaron diferentes términos para referirse a sus hijos además del término superviviente, estos incluyeron “ganador”, “victorioso” y “guerrero”. Vivir después de un “diagnóstico de muerte” es una característica importante para definir a los superviviente desde la perspectiva de los padres.

2. Subtema: “Es un renacimiento”: Al igual que en la guerra, donde la supervivencia es incierta, el tratamiento del cáncer es como “una experiencia cercana a la muerte”. Debido a la cantidad de riesgos potenciales para la vida que se atraviesan durante los “tiempos de guerra”, la supervivencia se vive como un milagro, como un renacimiento para el superviviente. Los padres también consideraban que habían renacido después del diagnóstico y tratamiento del cáncer de sus hijos. También manifestaron que tenían nuevas expectativas y prioridades de vida después de la difícil experiencia: *Es una nueva vida. Nació de nuevo; Nací de nuevo. Yo tenía otra expectativa sobre la vida, ahora es otra vida, totalmente diferente (Madre, p8).*

Los padres expresaron su agradecimiento por el trato que recibieron en el hospital y, según ellos, el hospital le devolvió la vida a su hijo y les permitió sobrevivir al cáncer infantil.

3. Subtema: Alianzas para lograr tiempos de paz: En tiempos de guerra, es necesario formar alianzas para lograr la paz, alianzas que ayuden a ganar cada batalla necesaria para lograr sobrevivir. Las alianzas provienen de recursos como la espiritualidad, la familia y los amigos. La espiritualidad, la fe y la esperanza se utilizaron como recursos para afrontar la situación, ayudaron a los padres a creer que llegarían tiempos de paz. Otro recurso mencionado fue la familia. Según uno de los padres, “para ser un superviviente, tu familia no debe darse por vencida”. Una madre mencionó que el cáncer es una enfermedad familiar y que afecta a toda la unidad, por lo tanto, toda la familia es superviviente del cáncer infantojuvenil.
4. Subtema: “La madre de un superviviente nunca se siente aliviada”: Al igual que en la guerra, cuando se anuncia su fin, todavía quedan dudas sobre los tiempos de paz. Mantener la paz requiere un control constante y estar siempre atento a los signos y síntomas de una posible nueva guerra. Los padres describieron el miedo a la recidiva como un factor que perturbaba constantemente la paz: *Lo que aún me afecta es el miedo a la recidiva. La madre del superviviente nunca más se siente aliviada (Madre, p5).*

Por lo tanto, dudaban de los tiempos de paz, a veces en silencio y soledad, los padres no compartían sus miedos. El miedo a que vuelva la guerra es un miedo que ocultan, para preservar la unión y la paz familiar y, por eso, no lo comparten con otros miembros, ni siquiera con el superviviente. El miedo a la recidiva se describió como un factor que afecta la calidad de vida de los padres y que, de los dos, la madre es la más afectada. La paz en la supervivencia del cáncer infantil es incierta, no sólo por la posibilidad de que se produzcan nuevos síntomas de recidiva, sino también por los traumas que deja la guerra. *Cada vez que tienes miedo y algo te asusta. Intentas hacer una vida normal como antes, pero no puedes. Siempre es como, “Dios mío, ¿está aquí otra vez?” Hoy estoy más atenta a cada movimiento que ella hace, cada pequeña señal es una advertencia y por eso hoy estoy más atenta (Madre, p3).*

## Discusión

El presente estudio proporcionó una comprensión profunda de las experiencias de los padres de supervivientes del cáncer infantil, al relacionar su experiencia en tiempos de guerra y en tiempos de paz incierta. El primer tema presentó el recorrido que realizaron los padres para obtener un diagnóstico adecuado para su hijo, en ocasiones los profesionales de la salud confundieron los síntomas que presentaba el paciente con otros problemas de salud, retrasando años algunos diagnósticos. La atención de salud brasileña es claramente heterogénea y muchas regiones del país aún carecen de capacitación profesional relacionada con la oncología pediátrica y de infraestructura adecuada, lo que dificulta la detección temprana del cáncer en la infancia. Los servicios de atención especializada en cáncer se concentran más en las grandes ciudades y regiones más desarrolladas del país, lo que también contribuye a la desigualdad en el tratamiento del cáncer infantojuvenil^34^. Por lo tanto, estas diferencias regionales en la prestación de dichos servicios hacen que las tasas de supervivencia en el país aún est**é**n por debajo de las esperadas para el desarrollo y el conocimiento técnico actuales. Esto ocurre porque cuestiones inherentes a la organización del sistema de salud y al acceso oportuno al diagnóstico contribuyen a determinar diferentes posibilidades de supervivencia^34^ ^)-(^ ^35^.

Los resultados mostraron que los padres y sus hijos enfrentan desafíos multifacéticos en el diagnóstico, tratamiento y supervivencia del cáncer infantil. La experiencia vivida tiene un impacto profundo en sus vidas, que forja una nueva identidad después del cáncer, y no les permite volver a ser los mismos que eran antes de la enfermedad. Nueve de los diez padres que participaron en el estudio describieron a sus hijos como supervivientes de cáncer infantojuvenil y también consideraron que tanto ellos como su familia se identificaban como “superviviente”. La definición de superviviente de cáncer del Instituto Nacional del Cáncer (INCA) incluye a familiares, amigos y cuidadores, ya que ellos también se ven afectados por el cáncer^10^ ^),(^ ^36^. Sin embargo, uno de los padres de este estudio no identificó a su hijo y su familia como supervivientes y de la misma manera, la literatura muestra que en realidad no todos se identifican con este término^37^ ^)-(^ ^38^. La evidencia indica que el no considerarse superviviente de cáncer a menudo se debe al curso del tratamiento (por ejemplo, no requerir quimioterapia ni radiación), a un pronóstico favorable y a la confianza en quienes brindan atención oncológica^39^ ^)-(^ ^40^.

Vivir después del cáncer es una experiencia constantemente “disruptiva”^41^ e incluso en la supervivencia a largo plazo, es posible que los niveles de estrés que desencadenaron el diagnóstico y el tratamiento en los padres no disminuyan^42^ ^)-(^ ^43^. Los resultados de este estudio demostraron que el miedo que experimentan los padres afecta su tranquilidad en la supervivencia y también su calidad de vida. La evidencia demuestra que los padres de supervivientes de cáncer infantil tienen altos niveles de sufrimiento psicológico, como síntomas de estrés postraumático, depresión, ansiedad, trastornos del sueño, somatización, miedo a la recidiva, gran preocupación y fatiga^42^ ^)-(^ ^44^. La incertidumbre después del tratamiento y la necesidad de lidiar con lo desconocido llevan a los padres a un ciclo de hipercontrol y, por ende, al sufrimiento continuo^45^. Al igual que los resultados de este estudio, la literatura muestra que las madres son los miembros de la familia que se sienten más afectados por el sufrimiento psicológico^46^ ^)-(^ ^47^.

Sin embargo, en las narrativas indican que los padres creían que, después del “renacimiento”, eran mejores personas, al igual que a sus hijos, y que tenían nuevas expectativas de vida. También se pueden registrar resultados positivos después de un evento estresante, como el diagnóstico de cáncer infantojuvenil^48^, proceso definido en la literatura como crecimiento postraumático^48^ ^)-(^ ^49^. El crecimiento postraumático implica los procesos de creación de nuevos significados después de un evento traumático, que pueden incluir el descubrimiento de beneficios y el desarrollo personal^48^ ^)-(^ ^49^.

Este estudio tiene puntos fuertes que incluyen el método narrativo, descripción rigurosa del reclutamiento de los participantes, entrevistas semiestructuradas, exhaustivas y densas, análisis riguroso de los datos con dos codificadores independientes y construcción de temas y subtemas con todo el equipo de investigación. Además, los resultados presentados en este estudio aclaran la experiencia de ser padre de un superviviente de cáncer infantojuvenil e indican que es necesario que se realicen futuros estudios, y que los profesionales de la salud y los medios de comunicación deben ser sensibles a esta experiencia, que aún es poco comprendida en la literatura. Sin embargo, se reconoce que el estudio tiene limitaciones. Realizar una sola entrevista con los padres puede haber limitado la profundidad de la comprensión de aspectos importantes de la experiencia descrita por los participantes en la primera entrevista. Los padres fueron incluidos en dos fases distintas de la supervivencia del cáncer infantojuvenil, extendida y permanente, lo que puede haber limitado la comprensión de la particularidad de la experiencia de ser padre en cada una de estas etapas de supervivencia. Además, si bien en otros estudios que incluyen a los padres, la representación de las madres también es mayor que la de los padres, hay que considerar una limitación el haber incluido a un solo padre en la muestra de este estudio.

El presente estudio allana el camino para futuras investigaciones con padres de supervivientes de cáncer infantil. Es importante que estudios futuros exploren la experiencia con diferentes estructuras familiares y contextos culturales de los participantes incluidos en este estudio, para discutir mejor las narrativas de los padres aquí descritas y agregar nuevos conocimientos sobre su experiencia durante la supervivencia. Aunque el estudio se desarrolló durante la pandemia de COVID-19, nuestro objetivo no era explorar la influencia de la pandemia de COVID-19 en las experiencias de esos padres. Estudios cualitativos futuros pueden explorar la influencia de la pandemia de COVID-19 en la experiencia de los padres de supervivientes de cáncer infantojuvenil. Además, es necesario que futuras investigaciones desarrollen modelos de atención para supervivencia centrados en la familia con el objetivo de minimizar el impacto del cáncer en sus vidas. El modelo actual de atención para la supervivencia del cáncer en niños y adolescentes aún no incluye a la familia, el foco está en el paciente. En el área de la educación, la perspectiva presentada en los resultados de este estudio puede orientar la enseñanza de grado y posgrado, y formar a los profesionales de la salud para ser más sensibles a las particularidades de las experiencias de los padres de supervivientes de cáncer infantojuvenil. Investigaciones futuras también podrían buscar posibles diferencias entre las experiencias de las madres y los padres de supervivientes de cáncer infantojuvenil. Ese es un dato interesante para que los enfermeros puedan adaptar sus cuidados, teniendo en cuenta también la cuestión del género en los cuidados de enfermería además del cáncer.

Por último, los resultados de este estudio aportan una serie de implicaciones clínicas al ámbito de la enfermería, independientemente del escenario de actuación, atención especializada o básica. En primer lugar, es necesario que los gestores de salud inviertan en cualificaciones profesionales que permitan el reconocimiento oportuno de los signos y síntomas del cáncer infantil y, por ende, reducir el tiempo de diagnóstico. Las enfermeras pueden identificar signos y síntomas de cáncer en niños y adolescentes, y contribuir para que accedan antes a la atención médica. Como se analizó anteriormente, el diagnóstico oportuno aumenta las tasas de supervivencia del cáncer infantojuvenil. En este estudio, los padres utilizaron metáforas para informar acerca de sus experiencias con respecto a la supervivencia de sus hijos al cáncer infantojuvenil. Las metáforas pueden contribuir a la atención de enfermería cualificada, centrada en el paciente y la familia, ya que ayudan a traducir la experiencia de la enfermedad y hacerla accesible al superviviente y a sus padres^50^. El sistema de metáforas también puede contribuir a la creación de otros significados y conceptos relacionados con el cáncer, que aún está estigmatizado y es visto como un diagnóstico de muerte. Sin embargo, debe utilizarse con respeto y sensibilidad, observando la forma de comunicación elegida por la familia y el paciente. Los resultados también demostraron que el miedo a la recidiva tiene un impacto en la paz de los padres, que incluye la calidad de vida de los mismos. Los profesionales de la salud pueden considerar la importancia que tiene brindar espacios para que los padres expresen sus miedos y sentimientos, así como también información relacionada con la recidiva del cáncer infantojuvenil y, así, reducir el impacto del miedo en sus vidas. Desafortunadamente, gran parte de la atención de supervivencia al cáncer infantojuvenil se centra en la atención clínica, y no se le da la importancia al enfoque psicoemocional.

## Conclusiones

Los resultados de este estudio aportan una nueva perspectiva que muestra que la experiencia de los padres durante la supervivencia del cáncer infantojuvenil está permeada por tiempos de guerra y tiempos de paz incierta. Los resultados de esta investigación pueden brindar una comprensión más profunda y coherente de las experiencias de los padres de supervivientes de cáncer infantojuvenil en la supervivencia prolongada y permanente, así como también ayudar a diseñar mejores cuidados de enfermería basados en evidencia para esta población. Por otro lado, las enfermeras pueden utilizar este conocimiento para desarrollar modelos de atención centrados en la familia para los supervivientes y mejorar la calidad de vida a largo plazo de los padres y de los supervivientes.
